# Promoting ethylene production over a wide potential window on Cu crystallites induced and stabilized via current shock and charge delocalization

**DOI:** 10.1038/s41467-021-27169-9

**Published:** 2021-11-24

**Authors:** Hao Sun, Ling Chen, Likun Xiong, Kun Feng, Yufeng Chen, Xiang Zhang, Xuzhou Yuan, Baiyu Yang, Zhao Deng, Yu Liu, Mark H. Rümmeli, Jun Zhong, Yan Jiao, Yang Peng

**Affiliations:** 1 Soochow Institute for Energy and Materials Innovations, College of Energy, Soochow University, 215006 Suzhou, P. R. China; 2 Key Laboratory of Advanced Carbon Materials and Wearable Energy Technologies of Jiangsu Province, Suzhou, P. R. China; 3 School of Chemical Engineering and Advanced Materials, The University of Adelaide, Adelaide, SA 5005 Australia; 4 Institute of Functional Nano & Soft Materials (FUNSOM), Jiangsu Key Laboratory for Carbon-Based Functional Materials & Devices, Soochow University, 215123 Suzhou, China

**Keywords:** Electrocatalysis, Electrocatalysis, Electrocatalysis

## Abstract

Electrochemical CO_2_ reduction (CO_2_RR) in a product-orientated and energy-efficient manner relies on rational catalyst design guided by mechanistic understandings. In this study, the effect of conducting support on the CO_2_RR behaviors of semi-conductive metal-organic framework (MOF) — Cu_3_(HITP)_2_ are carefully investigated. Compared to the stand-alone MOF, adding Ketjen Black greatly promotes C_2_H_4_ production with a stabilized Faradaic efficiency between 60-70% in a wide potential range and prolonged period. Multicrystalline Cu nano-crystallites in the reconstructed MOF are induced and stabilized by the conducting support via current shock and charge delocalization, which is analogous to the mechanism of dendrite prevention through conductive scaffolds in metal ion batteries. Density functional theory calculations elucidate that the contained multi-facets and rich grain boundaries promote C–C coupling while suppressing HER. This study underlines the key role of substrate-catalyst interaction, and the regulation of Cu crystalline states via conditioning the charge transport, in steering the CO_2_RR pathway.

## Abstract (web summary)

The substrate-catalyst interaction plays a critical role in steering the pathway of electrochemical CO_2_ reduction. Here the authors show the crystalline states of copper in reconstructed metal-organic frameworks were induced and stabilized by Ketjen Black conducting support, promoting C_2_H_4_ production.

## Introduction

The escalating climate change originating from the greenhouse effect due to excessive CO_2_ emission urges the mankind to realize carbon neutrality as early as possible^1,2^. Among the multiple strategic route maps, electrocatalytic reduction of carbon dioxide (CO_2_RR) driven by renewable energy is especially tempting, as it simultaneously addresses both the environmental and resource crises^3,4^. Copper in various forms have been demonstrated highly potent in catalyzing CO_2_ conversion into a diversity of hydrocarbons and oxygenates, owing to its modest binding to many of the key CO_2_RR intermediates^5,6^. This, however, also leads to the lack of product selectivity in Cu-catalyzed CO_2_RR as a result of the scaling relation among those intermediate binding^7,8^. To take advantage of the product diversity while maximizing the selectivity of individual target, many structural mutations of Cu have been explored, including morphology and facet control^9,10^, creation of grain boundaries^11,12^, under-coordinated sites^13,14^, and mixed valence states^15,16^, as well as surface functionalization and segregation^17,18^. Nonetheless, energy efficiency and product selectivity, especially on those value-added multiple-carbon products, remain as the major hurdles for ultimately deploying the technology in a techno-economic fashion.

Previous studies showed that the substrates for catalyst loading, in most cases the carbon support, play a critical role in steering the CO_2_RR pathway and stabilizing the catalytic process^19,20^. For instance, Strasser and colleagues^21^ showed that the dispersion of Cu_2_O nanocubes on a carbon support sharply shifted the selectivity pattern toward C_1_, whereas its unsupported counterpart achieved a sustained C_2+_ Faradaic efficiency (FE). In situ formation of small Cu seeds with larger interparticle distance were attributed to the witnessed C_1_ selectivity on the supported Cu_2_O. Differently, Hwang and colleagues showed that Cu_2_O nanoparticles immobilized on a carbon support via cysteamine doubled the FE of C_2_H_4_ in comparison to the unsupported catalyst and ascribed this selectivity enhancement to the electrochemical fragmentation of Cu_2_O nanoparticles into compactly stacked smaller particles that are more liable to oxidation^22^. In another study, by pre-reducing the Cu nodes in a conductive metal organic framework (MOF) (CuHHTP) into Cu_2_O quantum dots, Cao and co-workers achieved a high selectivity of CH_4_ formation, which was attributed to ample hydrogen bonding from the substrate in stabilizing key intermediates^23^. Thus, it is vitally important to assess the effect of carbon support on CO_2_RR and scrutinize the substrate–catalyst interaction, as well as the charge transport behavior, for a better mechanistic understanding.

**Fig. 1:**
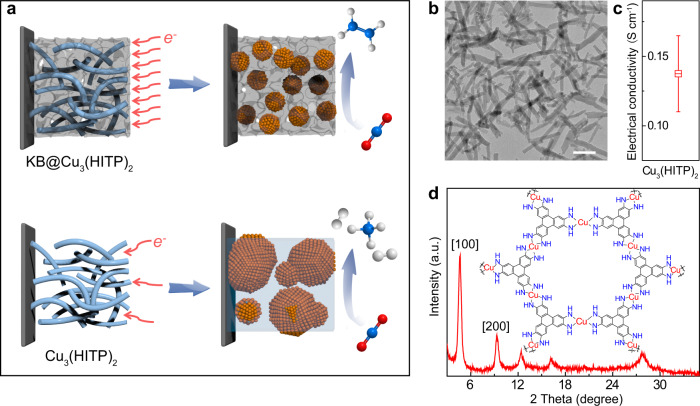
Schematic representation and structure characterization. **a** Schematic diagrams showing the modulation of CO_2_RR product selectivity by Cu_3_(HITP)_2_-derived Cu^0^ entities in the presence (top) or absence (bottom) of carbon support. **b** TEM image, **c** electrical conductivity measured by the four-probe method, and **d** XRD pattern of the as-synthesized Cu_3_(HITP)_2_. The scale bar in **b** is 200 nm. Error bars represent the standard deviation of three independent measurements.

MOFs have been perceived as a unique category of CO_2_RR catalyst since they offer a tunable platform to systematically alter the metal site coordination^24^, regulate CO_2_ and electrolyte counterions in the Helmholtz layer^25^, and control over the intermediate binding^26^. However, the stability of MOFs during the electrolytic process has been always a limiting issue, so that their electrochemical reconstruction (which is almost unavoidable, especially under high current conditions) to derive more robust catalyst ensembles is gaining increasing attentions^27,28^. Consequently, when the controllable electrochemical reconstruction of MOFs is the subject of study, their chemical stability in the electrolyte is a premise^29^. For that, in the current study we set to investigate the CO_2_RR behaviors of a semi-conductive MOF—Cu_3_(HITP)_2_ (HITP = 2,3,6,7,10,11-hexaiminotriphenylene) with and without adding the conducting support of Ketjen Black (KB). Cu_3_(HITP)_2_ was synthesized in an alkaline environment and thus, unlike many carboxylic MOFs, was born chemically resilient to the common electrolytes of CO_2_RR. This should endow a unique opportunity to allow us to focus on their electrochemical reconstruction and catalyst–support interaction during the electrochemical process (Fig. 1a). We found that adding KB enabled greatly promoting the C_2_H_4_ production with stabilized FE between 60 and 70% in a wide potential range and prolonged testing period, whereas the stand-alone MOF yielded more mixed reduction products. Operando X-ray absorption spectroscopy (XAS), serial post-reaction X-ray diffraction (XRD), and transmission electron microscope (TEM) analyses in conjunction with density functional theory (DFT) calculations were further carried out to correlate the product selectivity with structural characteristics of the reduced Cu moieties, furnishing fresh insights into the substrate–catalyst interaction and promoted C–C coupling on multicrystalline Cu nano-crystallites.

## Results

### Structural characterization of Cu_3_(HITP)_2_

Cu_3_(HITP)_2_ with high crystallinity was synthesized following the method developed by Dincă and co-workers recently^30^. TEM taken on Cu_3_(HITP)_2_ revealed rod-like crystals of 30–40 nm in diameter (Fig. 1b), while energy-dispersive X-ray elemental mapping by high-angle annular dark-field scanning TEM confirmed the homogeneous elemental distribution of Cu, N, and C (Supplementary Fig. 1). Owing to the in-plane electron delocalization and through-space charge transport^31^, Cu_3_(HITP)_2_ demonstrated a semi-conductivity of 0.14 S cm^−1^ (Fig. 1c), as measured at room temperature by the four-point probe method and in line with the previous reported values^31^. The XRD pattern in Fig. 1d exhibiting prominent diffraction peaks between 3° and 30° well matched that previously reported for Cu_3_(HITP)_2_^30–32^, affirming the successful synthesis of the MOF structure with high crystallinity. X-ray photoelectron spectroscopy of Cu 2p_3/2_ exhibited a mixture of Cu^2+^ (933.5 eV) and Cu^+^ (931.8 eV) states in Cu_3_(HITP)_2_ (Supplementary Fig. 2)^31^, which was further corroborated by the X-ray absorption near edge structure (XANES, Supplementary Fig. 3). While the main peak at 398.5 eV in the N 1s spectrum was attributed to the Cu-N motif (Supplementary Fig. 4)^31^, the shoulder at 396.7 eV corresponded to the decrease in the N valence caused by the uncoordinated amines at the edge or defect sites of Cu_3_(HITP)_2_^33^.

### CO_2_RR of Cu_3_(HITP)_2_ with or without KB

Considering the alkaline stability and intrinsic conductivity of Cu_3_(HITP)_2_, electrochemical CO_2_RR performance was evaluated without or with adding KB as the conducting agent in CO_2_-saturated KHCO_3_ electrolyte. Correspondingly, the catalysts were denoted as Cu_3_(HITP)_2_ and KB@Cu_3_(HITP)_2_, respectively. First of all, linear sweep voltammetries (LSVs, with a rate of 50 mV s^−1^) taken on KB@Cu_3_(HITP)_2_ showed notably higher current densities than those of Cu_3_(HITP)_2_ in both N_2_- and CO_2_-purged electrolytes (Supplementary Fig. 5), indicating the addition of KB helped to promote the Faradaic processes including both hydrogen evolution reaction (HER) and CO_2_RR. In addition, both KB@Cu_3_(HITP)_2_ and Cu_3_(HITP)_2_ gave higher current densities under CO_2_RR conditions, affirming that the Cu-containing MOF was effective in catalyzing electrochemical CO_2_ reduction. When normalized to the electrochemically active surface area estimated by measuring the double-layer capacitance (*C*_dl_), the LSV current densities of KB@Cu_3_(HITP)_2_ were still higher than those of Cu_3_(HITP)_2_ (Supplementary Fig. 6), suggesting the intrinsic higher catalytic activity of the former.

**Fig. 2:**
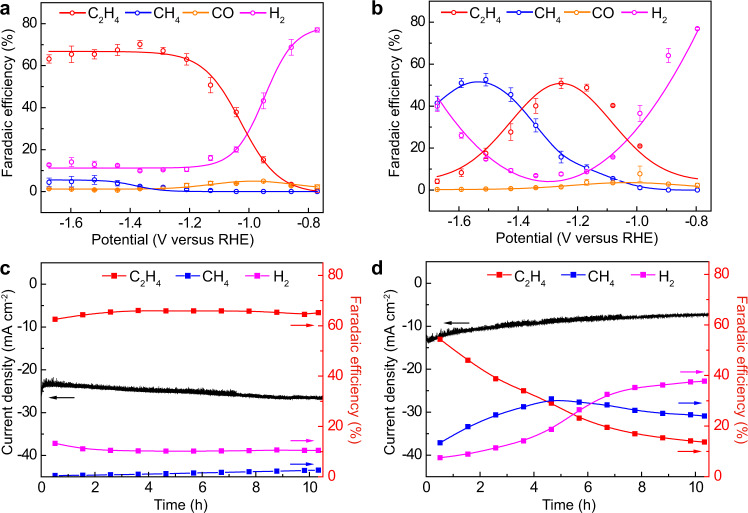
Electrochemical CO_2_RR performances. FEs of C_2_H_4_, CH_4_, CO, and H_2_ on **a** KB@Cu_3_(HITP)_2_ and **b** Cu_3_(HITP)_2_. The 10-h chronoamperometric test at −1.25 V for **c** KB@Cu_3_(HITP)_2_ and **d** Cu_3_(HITP)_2_ showing the evolution of total current density and FEs of C_2_H_4_, CH_4_, and H_2_. Error bars represent the standard deviation of three independent measurements.

On analyzing the reduction products, it was impressive to see KB@Cu_3_(HITP)_2_ exhibiting a higher and more stable C_2_H_4_ selectivity and yield (Fig. 2a, b and Supplementary Fig. 7). For KB@Cu_3_(HITP)_2_, the production of C_2_H_4_ started at −0.85 V (reversible hydrogen electrode (RHE), hereafter all the potential mentioned are referenced to this format) and reached a maximum FE of 70% at −1.37 V with concomitantly decreased H_2_ yield (Fig. 2a). Of note, the FEs of C_2_H_4_ maintained over 63% within the wide potential range from −1.20 to −1.67 V, whereas those of CH_4_ and CO were trivial during the entire CO_2_RR test. In comparison, the FEs of C_2_H_4_ for Cu_3_(HITP)_2_ displayed a maximum of 51% at −1.25 V and thereafter decreased continuously (Fig. 2b). Meanwhile, a notable CH_4_ production was observed attaining its maximum FE of 53% at −1.51 V, accompanied by a continuous increase in H_2_ yield (Fig. 2b). The different CO_2_RR behaviors between KB@Cu_3_(HITP)_2_ and Cu_3_(HITP)_2_, especially on the varied C_2_H_4_ and CH_4_ production, implied a different structural mutation of the Cu_3_(HITP)_2_ MOF during CO_2_RR, which will be closely inspected later.

In addition to the C_2_H_4_ FE of >60% in a wide potential range, KB@Cu_3_(HITP)_2_ also demonstrated a high partial current density reaching 37.4 mA cm^−2^ at −1.67 V (Supplementary Fig. 7), which is seldom seen in H cells (Supplementary Table 1). Moreover, at the applied potential of −1.25 V, KB@Cu_3_(HITP)_2_ showed a good CO_2_RR stability, with the total current density increased slightly from 23.3 to 26.3 mA cm^−2^ and C_2_H_4_ FE constantly above 64% for a duration of 10 h (Fig. 2c). By contrast, in the same testing period the C_2_H_4_ FE of Cu_3_(HITP)_2_ decreased continuously from 52 to 13%, concomitant with a drop of total current density from 13.3 to 7.2 mA cm^−2^ (Fig. 2d). As for CH_4_ production, it was intriguing to see both its FE and partial current density on Cu_3_(HITP)_2_ were higher than those on KB@Cu_3_(HITP)_2_, suggesting that the former was less competent in triggering C–C coupling. The liquid reduction products, analyzed by nuclear magnetic resonance (NMR), were also collected for both KB@Cu_3_(HITP)_2_ and Cu_3_(HITP)_2_, revealing a mixture of formate, ethanol, and propanol with a total FE <30% at all potentials (Supplementary Fig. 8). Thus, our focus of this study will be mainly placed on comparing the main gaseous products.

### Operando XAS on the Cu states

**Fig. 3:**
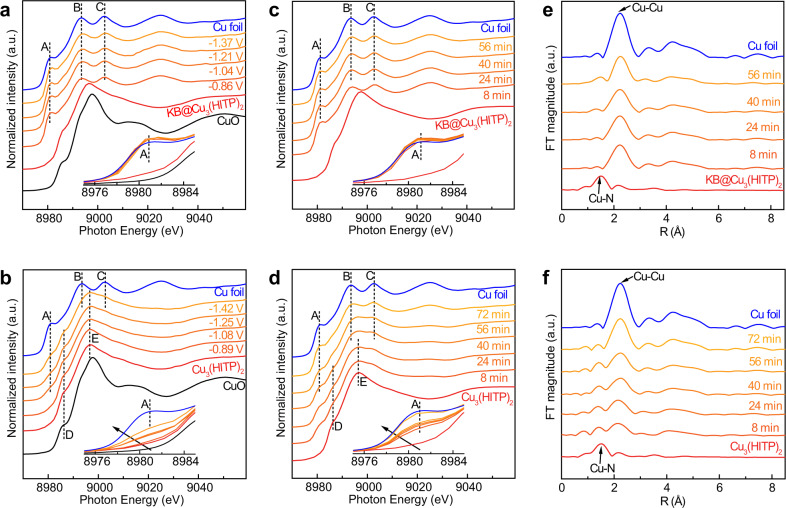
Operando XAS. Operando Cu K-edge XAS of **a** KB@Cu_3_(HITP)_2_ and **b** Cu_3_(HITP)_2_ at different potentials during CO_2_RR. Operando Cu K-edge XAS of **c** KB@Cu_3_(HITP)_2_ and **d** Cu_3_(HITP)_2_ as a function of reaction time at −1.25 V. The corresponding time-dependent FT-EXAFS for **e** KB@Cu_3_(HITP)_2_ and **f** Cu_3_(HITP)_2_.

To track the change of Cu states for KB@Cu_3_(HITP)_2_ and Cu_3_(HITP)_2_ during CO_2_RR and elucidate the origin of their catalytic difference, both potential- and time-dependent operando XAS were carried out by looking into the Cu K-edges. For KB@Cu_3_(HITP)_2_ (Fig. 3a), upon the application of a negative potential at −0.86 V, the positive Cu^*δ*+^ (1 < *δ* < 2) state of the MOF turned into Cu^0^, showing consistent pre-edge and white-line features (marked A, B, C in the XANES spectra of Fig. 3a) to those of Cu foil. In stark contrast, Cu_3_(HITP)_2_ showed a more gradual change of Cu states as the applied potential decreased from −0.86 to −1.37 V (Fig. 3b). While the pre-edge and white-line peaks common to Cu^2+^ in CuO and Cu_3_(HITP)_2_ (marked as D and E in Fig. 3b) were still retained at −1.42 V, those of Cu^0^ also started to evolve at the applied potential of −1.08 V, especially for the absorption edge at 8981 eV (feature A, due to the 1s → 4p_*z*_ transition)^34^. Clearly, these observations indicated that the Cu^*δ*+^ state in KB@Cu_3_(HITP)_2_ was more readily reduced than that in Cu_3_(HITP)_2_, likely due to the high current density from KB addition.

Similarly, the time-dependent XANES spectra of KB@Cu_3_(HITP)_2_ collected at a constant potential of −1.25 V showed the Cu^*δ*+^ state in KB@Cu_3_(HITP)_2_ was reduced to Cu^0^ within only 8 min (Fig. 3c), whereas that in Cu_3_(HITP)_2_ took 72 min to match the XANES spectrum of Cu foil (Fig. 3d). Furthermore, the serial Fourier-transform extended XAFS (FT-EXAFS) spectra of KB@Cu_3_(HITP)_2_ showed a rapid evolution of the metallic Cu-Cu bond at 2.2 Å and the disappearance of the MOF-related Cu-N peak at 1.5 Å within 8 min (Fig. 3e), corroborating that the Cu^*δ*+^ nodes in the MOF were quickly and fully reduced. In the next hour, the intensity of the Cu-Cu coordination peak did not change much, indicating that the as-formed Cu^0^ moieties remained mostly stable. On the other hand, the sequential FT-EXAFS spectra of Cu_3_(HITP)_2_ displayed a gradual decay of the Cu-N coordination but intensification of the Cu-Cu peak within the whole testing period of 72 min (Fig. 3f), attesting to slower change of the MOF structure with gradually reduced Cu states. Taken together from the above electrochemical and XAS studies, it becomes evident that a high current shock endowed by adding the more conductive KB enables to quickly reduce the Cu nodes in the semi-conductive MOF of Cu_3_(HITP)_2_.

### Ex situ time-lapse XRD and TEM

**Fig. 4:**
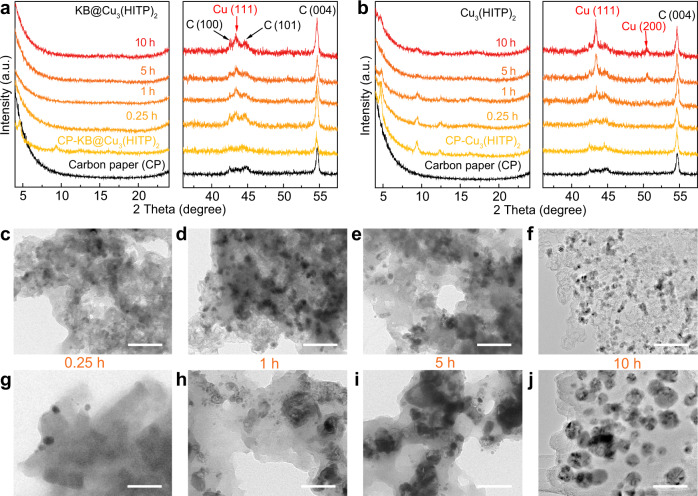
Ex situ time-lapse XRD and TEM. Sequential XRD patterns of **a** KB@Cu_3_(HITP)_2_ and **b** Cu_3_(HITP)_2_ at −1.25 V. TEM images taken on **c**–**f** KB@Cu_3_(HITP)_2_ and **g**–**j** Cu_3_(HITP)_2_ after a chronoamperometric testing period of 0.25, 1, 5, and 10 h at −1.25 V, respectively. The scale bars in **c**–**j** are 100 nm.

Ex situ time-lapse XRD and TEM were used to track the structural changes of KB@Cu_3_(HITP)_2_ and Cu_3_(HITP)_2_ under a fixed CO_2_RR potential of −1.25 V. As shown in the left panel of Fig. 4a, for KB@Cu_3_(HITP)_2_ the characteristic XRD peaks of the MOF disappeared completely after just 0.25 h of testing. In the meantime, a Cu(111) peak at 43.3° can be distinguished sandwiched between the C(100) and C(101) diffractions, and its intensity changed only marginally in the next 10 h (Fig. 4a, right panel). This observation is in good agreement with the previous XAS results, corroborating the quick reduction of Cu^*δ*+^ centers in the reticular network of the MOF to form metallic Cu species. By contrast, for the stand-alone Cu_3_(HITP)_2_, diffraction peaks specific to the MOF were still distinguishable after even a reaction period of 10 h (Fig. 4b, left panel), indicating that not all the catalyst was decomposed. Different from that of KB@Cu_3_(HITP)_2_, the intensity of the Cu(111) peak observed on Cu_3_(HITP)_2_ grew continuously along the reaction time, together with the gradual emergence of a Cu(200) peak at 50.4° (Fig. 4b, right panel). Again, the different trend of XRD evolution observed above on KB@Cu_3_(HITP)_2_ and Cu_3_(HITP)_2_ suggested that in the former the metallic Cu species were quickly formed and remained relatively stable, whereas in the latter Cu^*δ*+^ were reduced more gradually but aggregated into larger Cu particles of higher crystallinity (according to the Debye–Scherrer rule, Supplementary Fig. 9 and Table 2)^35^, which will be further examined by the following TEM studies.

Figure 4c–f, g–j displayed the TEM images taken on KB@Cu_3_(HITP)_2_ and Cu_3_(HITP)_2_ after a chronoamperometric testing period of 0.25, 1, 5, and 10 h at −1.25 V, respectively. The nano-rod morphology of the as-prepared Cu_3_(HITP)_2_ was absent in all post-electrolytic KB@Cu_3_(HITP)_2_ and Cu_3_(HITP)_2_ samples, indicating the corruption of the MOF topologic structure. Particularly, for KB@Cu_3_(HITP)_2_, Cu nanoparticles of 10 ± 2 nm were visible after 0.25 h, and beyond that the particle size remained mostly unchanged (Fig. 4c–f). As summarized in Supplementary Fig. 10a, the insignificant change of Cu particle size observed here coincided well with the stable C_2_H_4_ production seen in Fig. 2c. In contrast, for Cu_3_(HITP)_2_, the average size of Cu particles reduced out from the MOF increased from 11 ± 3 nm at 0.25 h to 39 ± 8 nm after 10 h (Fig. 4g–j), consistent with the above XRD observations (Fig. 4b and Supplementary Table 2). Correspondingly, the FE of C_2_H_4_ was reduced from the initial 52 to 13%, while that of H_2_ increased from 7.5 to 37% (Fig. 2d). This suggests that the growth of Cu particle size correlates to the decrease in C_2_H_4_ production and increase in H_2_ yield (Supplementary Fig. 10b). Similar evolution of Cu particles was also observed in the serial potential-dependent TEM images (Supplementary Fig. 11), showing the severe aggregation of Cu particles in Cu_3_(HITP)_2_ and mostly unchanged Cu particle size in KB@Cu_3_(HITP)_2_ while ramping down the potential from −1.21 to −1.75 V. Here, once again, the large Cu particle size in Cu_3_(HITP)_2_ correlated to the increase in H_2_ FE and decrease in C_2_H_4_ FE (Supplementary Fig. 12).

Collectively from the above observations, two facts about the CO_2_RR processes on KB@Cu_3_(HITP)_2_ and Cu_3_(HITP)_2_ become clear. First, the addition of KB caused the quick reduction of Cu^*δ*+^ nodes from the MOF into small Cu^0^ crystallites and helped stabilize them thereafter. Without the conducting support, the metal nodes were reduced more slowly but easily aggregated. We attribute this phenomenon mainly to the high current density and homogenized charge distribution on the electrode endowed by the conducting support. This is indeed analogous to the process of dendrite formation that is often observed on the anodes of metal ion batteries, where improved charge delocalization on the less-conductive solid–electrolyte interface (SEI) would greatly suppress dendrite growth^36,37^. Likewise, in the current case of Cu_3_(HITP)_2_ the gradually reduced Cu species tend to form aggregates due to inhomogeneous surface polarization and inadequate nuclei seeding, whereas for KB@Cu_3_(HITP)_2_ the conducting substrate can afford better charge transport to effectively reduce the metal nodes and dissipate localized charges for mitigating Cu agglomeration. Second, smaller crystallites of Cu reduced out from the MOF were more beneficial for C_2_H_4_ production^22,28^, whereas larger Cu particles tend to induce severe hydrogen evolution reaction. In between, a transition phase might be favorable for yielding CH_4_.

### Control studies with naked Cu nanoparticles

In order to see whether similar FE trends could also be observed for naked Cu nanocrystals when dispersed over a carbon support or whether it is related to the utilization of MOF precursors, we mixed commercial Cu nanoparticles (10–30 nm) with KB (denoted as KB@CuNPs) and tested their CO_2_RR activity in H-cell. With the same mass loading, KB@CuNPs exhibited lower total current densities than those of KB@Cu_3_(HITP)_2_ at all applied potentials (Supplementary Fig. 13), indicative of inferior catalytic activity. The FEs of C_2_H_4_ for KB@CuNPs reached a maximum of 48% at −1.56 V (Supplementary Fig. 14a), which is lower than that of KB@Cu_3_(HITP)_2_ (~65%) at the same potential. At lower potentials, KB@CuNPs showed higher H_2_ selectivity with FEs 3–4 times higher than those of KB@Cu_3_(HITP)_2_. In addition, the C_2_H_4_ selectivity of KB@CuNPs was unstable on the time scale, with the FE dropped from 47 to 11% within 8 h at the applied potential of −1.56 V (Supplementary Fig. 14b). Meanwhile, the FE of H_2_ increased from 39 to 74%. Such differences in activity and selectivity between KB@Cu_3_(HITP)_2_ and KB@CuNPs underline the value of using MOF precursors to derive active Cu moieties toward enhanced CO_2_RR performance. Based on this comparative study, we surmise that the crystalline structure of the MOF-derived Cu crystallites and their interaction with residual ligands both contribute to the observed performance enhancement. As part of the evidence, TEM images showed that the Cu nanoparticles in KB@CuNPs agglomerated into larger nanoparticles after 8 h of electrolysis (Supplementary Fig. 15), coinciding with the decrease in C_2_H_4_ and increase in H_2_ selectivity. By contrast, the Cu crystallites in KB@Cu_3_(HITP)_2_ did not agglomerate over time, which implies the possible tethering effect of the residual ligands in addition to the effect of KB-promoted charge delocalization. The agglomeration of Cu nanoparticles on carbon paper after prolonged CO_2_RR was also previously observed by Yang and colleagues^38^ Above all, our control study with naked Cu nanoparticles underscores the unique advantage of MOF precursors to derive highly active Cu motifs upon reconstruction.

### Mechanistic comprehension of the C_2_H_4_ selectivity on Cu nano-crystallites

**Fig. 5:**
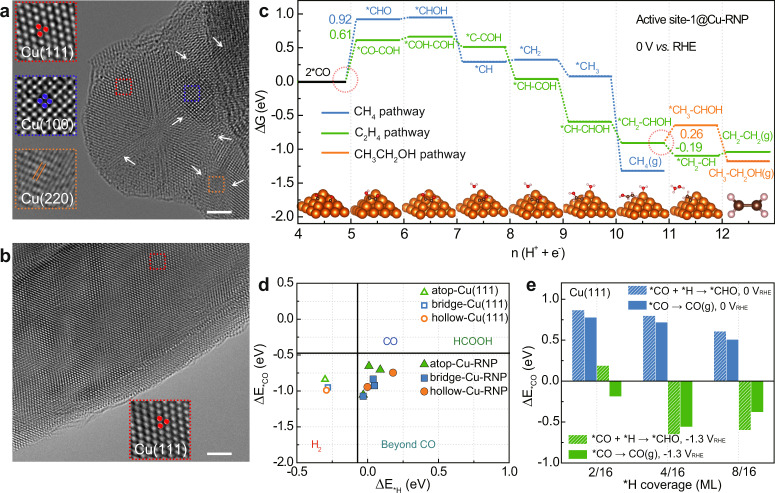
CS-TEM images and DFT simulation. TEM images of **a** KB@Cu_3_(HITP)_2_ and **b** Cu_3_(HITP)_2_ after CO_2_RR conducted at −1.25 V for 10 h in CO_2_-saturated 0.1 M KHCO_3_ electrolyte. **c** Reaction pathways starting with 2 *CO identified on active site-1 of [101] Cu rectangular nanopyramid (Cu-RNP) surface to model the post-electrolytic KB@Cu_3_(HITP)_2_ at 0 V. Insets are the atomic structures of intermediates along the C_2_H_4_ pathway. The key bifurcating points are highlighted with red circles and the unit of energy is eV. **d** Relation between ΔE_*CO_ and ΔE_*H_ at various sites on the Cu-RNP and Cu (111) surfaces. Vertical and horizontal black lines represent the equilibrium potential for *H ↔ ½ H_2_ and *CO ↔ CO, respectively and **e** the energetics of reactions *CO + *H → *CHO and *CO → CO (g) on the modeled post-electrolytic Cu_3_(HITP)_2_ surface of Cu (111) under various *H coverages and bias potentials. The scale bars in **a**, **b** are 2 nm.

To help further comprehend the observed structure–performance correlations from the aspect of atomic corrugations, we sought spherical aberration-corrected TEM (Cs-TEM) to dissect the lattice structure of the Cu nano-crystallites and nanoparticles derived from KB@Cu_3_(HITP)_2_ and Cu_3_(HITP)_2_, respectively, as well as DFT calculations to rationalize the associated C–C coupling process. Despite the much smaller particle size, Cu crystallites derived from KB@Cu_3_(HITP)_2_ after 10-h CO_2_RR test exhibited rich facets and grain boundaries, as well as lattice defects, in TEM images. For instance, as shown in Fig. 5a and insets^39^, various lattice planes of (111), (100), (220), etc. converged and were adjacent to each other, forming a highly complex lattice structure that are characteristic of pre-matured crystallites^40,41^. The small Cu crystallites were then stacked upon one and another, dispersed in the amorphous carbon matrix containing evenly distributed N elements that were inherited from the MOF ligands (Supplementary Figs. 16 and 17). On the other hand, the lattice structure of Cu particles reduced from Cu_3_(HITP)_2_ was relatively dull, containing less grain boundaries and defects. As shown in Fig. 5b and Supplementary Fig. 18, lattice fringes of the (111) plane with a *d*-spacing of 0.21 nm composed the main theme of the images, manifesting a more matured crystallinity. We attribute the observed difference in lattice diversity mainly to the relatively kinetics-driven Cu nucleation in KB@Cu_3_(HITP)_2_ but more of the thermodynamics-driven Cu growth in Cu_3_(HITP)_2_. That is to say, the as-formed Cu crystallites in KB@Cu_3_(HITP)_2_ were “frozen” in the earlier nucleation stage of crystal growth, where the packing of Cu atoms was more chaotic, leading to diverse lattice features^40^. In contrast, the reduced Cu nuclei in Cu_3_(HITP)_2_, with less population, tend to grow into lager conglomerates exposing less lattice features owing to Ostwald ripening^41^.

Learning the highly diverse lattice features of Cu nano-crystallites from post-electrolytic KB@Cu_3_(HITP)_2_ and the relatively dull lattice structure of Cu particles reduced from Cu_3_(HITP)_2_, we now stand on a good position to construct atomic models for simulating the CO_2_RR process. The difference in lattice configuration may result in non-identical electrochemical selectivity during CO_2_RR as suggested by previous research^42^. Therefore, in the following DFT study we focus on the effect of lattice configuration on the selectivity for C_2_ (e.g., ethylene) versus C_1_ (e.g., methane) production.

The post-electrolytic KB@Cu_3_(HITP)_2_ sample was modeled by [101] Cu rectangular nanopyramids (Cu-RNPs)^43^ as shown in Supplementary Fig. 19. The single Cu-RNP model is based on a rectangular 5 × 5 × 1 Cu (101) surface; the four triangular sidewalls of the nanopyramid are (100) and (111) facets at intervals. This model is in good agreement with Cs-TEM observation that (100) and (111) facets are converged and adjacent to each other (Fig. 5a). Moreover, the Cu-RNPs are in dense array with 5.1 Å between adjacent nanopyramids, which represents the small Cu crystallites stacked and dispersed on the substrate (Supplementary Fig. 16). Meanwhile, the post-electrolytic Cu_3_(HITP)_2_ surface with a uniform lattice structure was represented by a 4 × 4 × 4 Cu(111) supercell (Supplementary Fig. 20).

Three active sites were identified on Cu-RNP that evenly distribute on the symmetrical sidewalls (Supplementary Fig. 21). We then carried out DFT calculations to acquire the full reaction pathway as proposed by Cheng et al.^44^ toward main products observed in the experiment, including CH_4_ and C_2_H_4_. The reaction pathways are summarized in Fig. 5c and Supplementary Figs. 22 and 23, with free energy for each reaction step given in Supplementary Tables 3 and 4. From these reaction pathways, further reduction of 2*CO on Cu-RNP proceeds in two major pathways, namely, (1) C_1_ pathway (blue)—a conventional pathway toward CH_4_ formation and (2) C_2_ pathway which bifurcates from *CH_2_–CHOH to C_2_H_4_ (green) and C_2_H_5_OH (orange). The direct hydrogenation after *CO adsorption (2*CO + H^+^ + e^–^ → *CHO + *CO) has the highest endothermic energy in C_1_ pathway, serving as the potential-determining step (PDS). PDS for the C_2_ pathway is *CO dimerization and subsequent proton-coupled electron transfer (PCET) to *COH–CO (2*CO + H^+^ + e^–^ → *COH–CO). We also found that the limiting potential for C_2_ pathway on all active sites of Cu-RNP are significantly lower than those of C_1_ pathway (−0.61 to −0.68 V vs −0.84 to −0.92 V), which indicates that CO_2_RR can proceed preferentially toward C_2_ with an inhibited CH_4_ selectivity on Cu-RNP surface. In addition, for Cu-RNP C_2_ pathway, the proceeding of 11th PCET to *CH_3_–CHOH is endergonic with an uphill Δ*G* = 0.26 eV, in contrast to the exergonic protonation to *CH_2_–CH (Δ*G* = −0.19 eV), suggesting a low C_2_H_5_OH selectivity on the surface. All these are consistent with the experimental observation that on KB@Cu_3_(HITP)_2_ the FE for C_2_H_4_ production is significantly higher than those of CH_4_ and C_2_H_5_OH (Supplementary Fig. 8a).

The promoted C–C coupling and consequent high C_2_H_4_ selectivity on KB@Cu_3_(HITP)_2_ can be attributed to the enhanced *CO adsorption and geometric effect induced by rich grain boundaries and multi-facets on the reduced Cu nano-crystallites. As shown in Supplementary Fig. 24, the *CO binding on the boundary sites of Cu-RNP is significantly enhanced compared with those on basal sites and Cu(111). A strong *CO binding is highly sought after; for example, experimental efforts attribute the high C–C coupling activity to the strong *CO binding and the resulting high *CO coverage^45,46^. More importantly, on Cu-RNP along the grain boundaries, the strong *CO adsorption site and the adjacent weak adsorption site coexist within an under-coordinated surface square area (Supplementary Fig. 21), which meets *the extended square principles* criteria toward a promoted C–C coupling^43^. Consequently, the formation of *COH–CO on Cu-RNP is highly favored over that of *CHO and the C_2_ pathway is thermodynamically more selective than C_1_ pathway.

In addition to the promoted C–C coupling, the rich grain boundaries and multi-facets of Cu nano-crystallites on post-electrolytic KB@Cu_3_(HITP)_2_ can further contribute to higher C_2_ selectivity by inhibiting competitive HER. We adopted the selectivity determining method proposed by Rossmeisl and colleagues^6^ to indicate product selectivity on the constructed models by *H and *CO binding energy. The results are summarized in Fig. 5e, which shows that all active sites from the Cu(111) surface (Supplementary Fig. 25a) fall in the HER dominant zone. In contrast, all active sites from the Cu-RNP surface (Supplementary Fig. 25b) fall in the CO_2_RR dominant zone. The calculated selectivity on the two models is in good agreement with the experimental observation of stable C_2_H_4_ production with suppressed H_2_ yield on KB@Cu_3_(HITP)_2_.

It is worth noting that on Cu_3_(HITP)_2_ the selectivity of C_2_H_4_ starts to decrease from −1.3 V with a continuous increase in H_2_ and CH_4_ (Fig. 2b). We attribute the decline of C_2_H_4_ to the *H site-blocking effects under negative potentials. It was reported that dominant *H can partially displace *CO under very negative potential, and the resultant low *CO coverage impedes the *CO dimerization, hence C_2_H_4_ production^44^. We also observed that CH_4_ production attained the maximum FE of 53% after −1.0 V vs RHE with negligent amount of CO (Fig. 2b). The main reason lies in that Cu_3_(HITP)_2_ exposes mainly Cu(111) facets (Fig. 5b), which are more selective toward CH_4_ than CO under negative potentials according to previous experimental research^47,48^. To reveal the origin of CH_4_ selectivity over CO on Cu(111) surfaces, we simulated the key reaction step toward CH_4_ formation: *CO + *H → *CHO, and competing *CO desorption step: *CO → CO_(g)_ on Cu(111) (Supplementary Fig. 26). During the simulation, different electrode potentials (0 and −1.3 V vs RHE) were considered by applying the constant electrode potential (CEP) method^49^. At the same time, different surface *H coverages were considered (i.e., 2/16 ML, 4/16 ML and 8/16 ML), because previous experimental studies suggest that the coverage will increase to over 0.3 ML at high overpotential^50,51^. We found that, on Cu(111) at 0 V vs RHE, both the *CHO formation and *CO desorption activities improve from low *H coverage (2/16 ML) through medium *H coverage (4/16 ML) to high *H coverage (8/16 ML), as shown in Fig. 5e. With bias potential of −1.3 V vs RHE, both reaction steps become exergonic, while the former reaction is more thermodynamically favorable with more negative energy changes (∼−0.64 vs −0.38 eV). The difference in energy change means that the reaction pathway proceeds toward CH_4_ production at very negative potential via the *CHO formation, instead of CO release through *CO desorption. This conclusion is consistent with previous experimental research^47,48^, as summarized in Supplementary Table 5.

### Universality of the observations by extending to flow cell and Cu_3_(HHTP)_2_

**Fig. 6:**
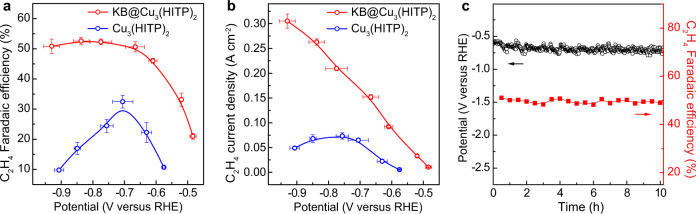
Electrocatalytic CO_2_RR performance in the flow cell. **a** FEs and **b** partial current densities of C_2_H_4_ at different potentials tested in the flow cell with 1 M KOH electrolyte for KB@Cu_3_(HITP)_2_ and Cu_3_(HITP)_2_. **c** Stability test of KB@Cu_3_(HITP)_2_ during 10 h of electrolysis under the current density of 300 mA cm^−2^. Error bars represent the standard deviation of three independent measurements.

As the CO_2_RR kinetics are restricted by mass transportation in the H cell due to limited solubility of CO_2_ in aqueous electrolytes^52^, flow-cell tests were further carried out in 1 M KOH to achieve high current densities (>100 mA cm^−2^, Supplementary Fig. 27). For a better quantification of FEs, the chronopotentiometric mode was employed. Despite the different electrolyte from that utilized in H cell, KB@Cu_3_(HITP)_2_ still showed notably higher current densities than those of Cu_3_(HITP)_2_ (Supplementary Fig. 28). In addition, the onset potential of C_2_H_4_ production for KB@Cu_3_(HITP)_2_ was −0.48 V, which is earlier than that for Cu_3_(HITP)_2_ (−0.58 V). For KB@Cu_3_(HITP)_2_, the FE of C_2_H_4_ reached 51% at −0.67 V and stayed above it till −0.93 V (Fig. 6a), whereas that for Cu_3_(HITP)_2_ displayed a maximum of 32% at −0.71 V but thereafter gradually decreased to below 5% at −1.02 V (Fig. 6a and Supplementary Fig. 29). As for CH_4_ production, the FE for KB@Cu_3_(HITP)_2_ was trivial and remained <9% during the entire CO_2_RR test, while that for Cu_3_(HITP)_2_ continued to increase with decreasing potentials until a maximum of 53% at −0.85 V (Supplementary Fig. 29). Besides, the presence of KB inhibited HER, especially at more negative potentials. Consistently, KB@Cu_3_(HITP)_2_ exhibited higher C_2_H_4_ partial current densities than Cu_3_(HITP)_2_ did at all applied potentials and reached 305 mA cm^−2^ at −0.93 V (Fig. 6b). KB@Cu_3_(HITP)_2_ also showed good electrocatalytic stability in the flow cell, which stably operated over 10 h at a current density of 300 mA cm^−2^ (Fig. 6c). In general, the flow-cell tests on KB@Cu_3_(HITP)_2_ and Cu_3_(HITP)_2_, despite involving much higher current densities, showed similar trends of product selectivity to those in the H cell, corroborating the positive role of KB in promoting and stabilizing the C_2_H_4_ selectivity.

To testify the universality of the above phenomenon with respect to the KB effect on promoting and stabilizing ethylene production for semi-conductive Cu MOFs that are stable in alkaline conditions, we synthesized another similar MOF of Cu_3_(HHTP)_2_ (HHTP = 2,3,6,7,10,11-hexahydroxytriphenylene) by replacing the amine groups in HITP with hydroxyls (Supplementary Figs. 30 and 31). As expected, similar CO_2_RR behaviors to that of Cu_3_(HITP)_2_ were also observed for Cu_3_(HHTP)_2_ (Supplementary Fig. 32). On KB@Cu_3_(HHTP)_2_, the FE of C_2_H_4_ was plateaued after −1.29 V (Supplementary Fig. 32a), coinciding with the stabilized Cu particle size at varying potentials from −1.22 to −1.65 V (Supplementary Fig. 33a–d). As for the stand-alone Cu_3_(HHTP)_2_, both the FEs of C_2_H_4_ and CH_4_ increased first and then decreased, concomitant with the “V”-shape voltage dependence of H_2_ FEs (Supplementary Fig. 32b), which was also observed previously for Cu_3_(HITP)_2_ (Fig. 2b). Correspondingly, the serial TEM images taken on the post-electrolytic Cu_3_(HHTP)_2_ samples at different potentials showed the prominent growth of Cu particle size with increasing bias (Supplementary Fig. 33e–h). Thus, for both Cu_3_(HITP)_2_ and Cu_3_(HHTP)_2_, the addition of KB enables steering the CO_2_RR pathway toward ethylene production by preserving the reduced Cu in their nano-crystallite state, which, as aforementioned, is induced and stabilized via high current shock and surface charge delocalization.

## Discussion

CO_2_RR behaviors of the semi-conductive MOF—Cu_3_(HITP)_2_—were studied with and without conducting support. It was found adding KB greatly promoted the C_2_H_4_ production with a stabilized FE between 60 and 70% in a wide potential range and prolonged test, whereas the stand-alone MOF produced more mixed reduction products during the reaction course. Operando XAS in conjunction with ex situ time-lapse XRD and TEM analyses clearly revealed that in the presence of KB tiny Cu crystallites were quickly reduced from the MOF and stabilized thereafter. By contrast, on Cu_3_(HITP)_2_ in the absence of KB, Cu nanoparticles were gradually reduced out and aggregated into larger sizes, which can be attributed to the poor surface charge delocalization, analogous to the process of dendrite growth at the SEI of metal ion batteries. By scrutinizing the detailed lattice structure using CS-TEM, a structural model comprising multi-facets and grain boundaries was established via DFT to successfully rationalize the promoted C–C coupling on the Cu nano-crystallites derived from KB@Cu_3_(HITP)_2_. As a result, our study furnishes fresh insights into the steering of CO_2_RR pathway by regulating and stabilizing Cu crystalline states via conditioning the charge transport on electrodes, which can be possibly extended to other metal–organic complexes.

## Methods

### Materials

Copper(II) sulfate pentahydrate (CuSO_4_·5H_2_O) was purchased from Sinopharm Chemical Reagent Co., Ltd. Triphenylene-2,3,6,7,10,11-hexaamine 6HCl (HATP·6HCl ≥98%) was provided by Shanghai Kaiyulin Co., Ltd. Triphenylene-2,3,6,7,10,11-hexaol (HHTP, ≥97%) was provided by Bide Pharmatech Ltd. Sodium acetate trihydrate (C_2_H_3_O_2_Na·3H_2_O, ≥99.5%) and cupric acetate anhydrous (Cu(C_2_H_3_O_2_)_2_) were obtained from Aladdin. Potassium bicarbonate (KHCO_3_, ≥99.5%) were purchased from J&K Scientific Co., Ltd. *N*,*N*-dimethylmethanamide (DMF, ≥99.5%) and *N*,*N*-dimethylacetamide (DMA, ≥99.5%) were purchased from Greagent. Ethanol (C_2_H_6_O, ≥99.7%) was provided by Shanghai Lingfeng Chemical Reagent Co., LTD. The CO_2_ gas (99.995%) was supplied by Suzhou Jinhong Gas Co. Ltd. All materials were used as received without further purification. The Cu NPs were purchased from Macklin. Deionized (DI) water was purified with a Sartorius arium mini ultrapure water system.

### Synthesis of Cu_3_(HITP)_2_

In all, 7 mg (0.028 mmol) of CuSO_4_·5H_2_O was suspended in 3 mL of DMA and sonicated for 10 min. Then a solution of 10 mg (0.0186 mmol) of HATP·6HCl in 3 mL of water was added and sonicated for 10 min. A total of 4 mL of C_2_H_3_O_2_Na aqueous solution (2 M) was then added at room temperature, and the mixture was heated in a 50 mL open glass vial with stirring for 2 h at 65 °C. The resulting black powder was filtered, washed with large amount of water and methanol, and dried under vacuum.

### Synthesis of Cu_3_(HHTP)_2_

In all, 28.3 mg (0.156 mmol) of Cu(C_2_H_3_O_2_)_2_ and 14 mg (0.044 mmol) of HHTP was suspended in 3 mL of DI water and sonicated for 5 min. A total of 0.3 ml DMF was then added dropwise to this mixture followed by 5 min of sonication. The capped vial was heated at 80 °C for 6 h. The resulting blue powder was centrifuged, washed with large amount of water and ethanol, and dried under vacuum.

### Characterizations

The crystalline phases of the products were analyzed by powder XRD measurements with a Bruker D8 Advance X-ray diffractometer using Cu-Ka radiation. One milligram of powder catalyst was loaded on 1 × 1 cm^2^ of carbon paper for ex situ time-lapse XRD testing. The surface composition and valence states were analyzed with XPS, using an Escalab 250Xi X-ray photoelectron spectrometer (Thermo Fisher) with Al Ka (1486.6 eV) X-rays as the excitation source, and the binding energy of the C 1s peak at 284.8 eV was taken as an internal reference. The pass energy was 30 eV and the photoemission angle was 45°. The energy linearity detection was calibrated with Au 4f (83.96 eV), Ag 3d5 (368.21), and Cu 2p (932.62 eV). The morphologies were examined by SEM conducted on a Hitachi SU8010 scanning electron microanalyzer with an accelerating voltage of 10 kV. The microstructures of the products were further characterized by TEM measured at 200 kV with a FEI TECNAI G20 field-emission TEM. Cs-TEM (FEI Titan Themis Cubed G2 300) was used to inspect atomic orientation of samples.

### Electrochemical measurements

The CO_2_ electro-reduction reactions were performed in a gas-tight, two-compartment H-cell controlled with an electrochemical workstation (CHI760E). Thereinto, the two compartments of H-cell are separated by an ion exchange membrane (Nafion perfluorinated membrane), and each compartment was filled with 40 mL of 0.1 M KHCO_3_. A carbon rod was used as the counter electrode, and a calibrated Ag/AgCl electrode was used as the reference electrode. To prepare the catalyst ink for H-cell studies, 4 mg of the sample powder and 1 mg KB were mixed with 50 µL Nafion solution (5%) in 1 mL ethanol by ultrasonic dispersion for 15 min. Next, 10 µL of the as-prepared ink was drop-coated onto the glassy carbon electrode with a surface area of 0.197 cm^−2^. The working electrode was then dried at room temperature for subsequent testing. Before electrochemical measurements, a continuous CO_2_ was purged into the cathodic compartment for at least 30 min. During the measurements, the CO_2_ was bubbled into the electrolyte with a constant flow rate of 20 cm^3^ min^−1^ controlled by a digital mass flow controller. The activation of the working electrode was carried out by repeatedly running cyclic voltammetry within the potential range from −0.5 to −1.8 V with a rate of 10 mV s^−1^ for 20 min.

The flow cell comprises three chambers: gas, catholyte (gas diffusion electrode, YLS-30T), and anolyte (nickel foam, 0.8 mm thickness). The Ag/AgCl reference electrode was plugged in the center of catholyte chamber via a top drill hole. Catholyte and anolyte were separated by anion exchange membrane (Fumasep FAB-PK-130). Gas and catholyte were separated by the gas diffusion electrode. Using an electric airbrush, the catalyst dispersed in ethanol was spray-coated on the side of the gas diffusion electrode facing the electrolyte (0.8 mg cm^−2^), with the opposite side facing the gas chamber. Silicone gaskets with 2 × 0.5 cm^2^ window were placed to assure adequate sealing of each chamber. The high-purity CO_2_ was supplied to the gas chamber with a constant flow rate of 30 cm^3^ min^−1^ monitored by a digital mass flow controller (Horiba). The flow-cell measurements were conducted in 1 M KOH with a constant flow rate of 20 ml min^−1^ through a dual-channel peristaltic pump. The pH of 1 M KOH decreased slightly from 13.7 to 13.3 after 10 h of reaction (Supplementary Fig. 34). The cell resistances were measured via electrochemical impedance spectroscopy under open circuit, and 85% ohmic resistance correction was applied in all H-cell and flow-cell measurements.

The gas products were quantitatively analyzed using gas chromatography equipped with both flame ionization and thermal conductivity detectors (Agilent 7890B). The liquid products were collected after at least 5 h of electrolysis and quantitatively analyzed using 1H NMR spectroscopy with H_2_O suppression. In all, 400 µL electrolyte mixed with 50 µL dimethyl sulfoxide (20 mM) and 100 µL D_2_O was used as the internal standard. All the potentials were converted to RHE, according to the equation *E* (vs RHE) = *E* (vs Ag/AgCl) + 0.059 × pH + 0.198.

### Operando XAS measurements

The operando XAS spectra at the Cu K-edge were recorded at the BL11B beamline of Shanghai Synchrotron Radiation Facility. The electron storage ring operated at 3.5 GeV. The beam current of the storage ring was 220 mA in a top-up mode. The incident photons were monochromatized by a Si(111) double-crystal monochromator, with an energy resolution Δ*E*/*E* ~ 1.4 × 10^−4^. The rejection of higher harmonics was achieved by a pair of Rh-coated mirrors at 4 mrad. The spot size at the sample was ~200 μm × 250 μm (*H* × *V*). The energy calibration was performed using a Cu foil. The operando XAS spectra were recorded in the fluorescence mode using a Lytle detector. The electrolysis was performed in CO_2_-saturated 0.1 M KHCO_3_ solution in a two-compartment H-shape cell. The sample was at 45° with respect to the incident beam direction. The electrodes were prepared as described in the “Electrochemical measurement” section. CO_2_ was bubbled into the cell with a constant flow rate during the operando experiments. Kapton tape was used to seal the cell. The operando XAS spectra were measured at a potential range from 0 to −1.42 V (keeping a constant potential when recording the spectrum). The running time for each spectrum was 8 min. XAS data were analyzed using the Athena software. All the XAS spectra were normalized to unity in Athena. The FT-EXAFS spectra were calculated between 3.0 and 11.0 Å^−1^ after weighting by *k*^3^.

### Computational methods

All DFT calculations were performed with the Vienna Ab Initio Simulation Package (VASP) code^53^. The Perdew–Burke–Ernzerhof was employed for electron exchange–correlation^54^. Projector Augmented Wave potentials were used to describe the ionic cores^55^. The atomic relaxations were carried out with the quasi-Newton minimization scheme until the maximum force on any atom was <0.03 eV Å^−1^. The geometry optimizations were performed with a plane-wave cutoff of 400 eV. Irreducible 2 × 3 × 1 or 2 × 2 × 1 Monkhorst Pack k-point grid was used^56^, with the center shifted to the gamma point. The Fermi level was smeared with the Methfessel–Paxton approach with a smearing of 0.1 eV. Dipole corrections were included in all the calculations to minimize the inaccuracies in the total energy due to the simulated slab interactions. The dipole moment was calculated parallel to the *z*-direction.

The implicit solvent effect was considered by using VASPsol^57^. The solvent dielectric constant was set to be 78.4, the width of dielectric cavity was 0.6 Å, the cutoff charge density was set to be 0.0025, and the effective cavity surface tension was 0.000525.

The lattice constant of Cu was optimized to be 3.64 Å in its fcc crystal structure. The Cu [101] RNPs were built based on a rectangular 5 × 5 × 1 Cu (101) surface; the four triangular sidewalls of the nanopyramid are (100) and (111) facets at intervals. The base layers were fixed while nanopyramids and adsorbates were permitted to fully relax in all configurations. The Cu (111) surfaces was constructed with 4 × 4 × 4 supercells, respectively, both with two bottom layers fixed. Two topmost layers and adsorbates were free to move in all directions. The vertical separation between periodically repeated images was set to be at least 15 Å in all cases, to ensure no interaction between images.

The free energies of adsorbed species, calculated based on the computational hydrogen electrode model^55^, are defined as:

1 $$\Delta {G}_{{{{{{\rm{ad}}}}}}}=\Delta {E}_{{{{{{\rm{ad}}}}}}}+{{{{{\rm{ZPE}}}}}}-T\Delta S$$

where ZPE is the zero-point energy correction and *T*∆*S* is the entropy correction, which were computed with standard methods^58^.

The formation free energy of *COH–CO (ΔG_*COH–CO_) is calculated as

2 $$\Delta {G}_{^{\ast}{{{{{\rm{COH}}}}}}-{{{{{\rm{CO}}}}}}}={G}_{{{{{{\rm{total}}}}}}}-({G}_{2^{\ast}{{{{{\rm{CO}}}}}}}+{G}_{{{{{{\rm{H}}}}}}2}/2)$$

where *G*_total_ is the free energy of a surface with adsorbed *COH–CO, *G*_2*CO_ is the free energy of a surface with two adsorbed CO molecules, and *G*_H2_ is the energy of a hydrogen molecule, which is −6.83 eV.

The formation free energy of *CHO (ΔG_*CHO_) is calculated as

3 $$\Delta {G}_{^{\ast}{{{{{\rm{CHO}}}}}}}={G}_{{{{{{\rm{total}}}}}}}-({G}_{^{\ast} {{{{{\rm{CO}}}}}}}+{G}_{{{{{{\rm{H}}}}}}2}/2)$$

where *G*_total_ is the free energy of a surface with adsorbed *CHO and G_*CO_ is the free energy of a surface with one adsorbed CO molecule. The free energy of relevant gas phases are listed in Supplementary Table 6.

Limiting potential (*U*_limiting_) is used to describe the lowest potential requirement to eliminate the free energy difference of the PDS, calculated as:

4 $${U}_{{{{{{\rm{limiting}}}}}}}=-\Delta G_{{{{{\rm{max}}}}}}/e$$

We applied the CEP model proposed by Head-Gordon and colleagues^49^ to consider the effect of applied potential on reactions. We simulated each surface with different electron numbers, i.e., −2|*e*|, 0|*e*|, and +2|*e*|. We then carried out potential calculation to obtain work function of each surface and corresponding electrode potentials. The electrode potential (*U*) of the slab relative to the normal hydrogen electrode (NHE) can be estimated from the work function, *φ*, relative to that of the NHE (*φ*_NHE_).

5 $$U=\varphi -{\varphi }_{{{{{{\rm{NHE}}}}}}}$$

where *φ*_NHE_ = 4.5 V as the work function of the reference system^59^. The linear relationship of electrode potentials and electron numbers on different models are presented in Supplementary Fig. 26. From Supplementary Fig. 26, different numbers of electrons were assigned to corresponding surfaces to simulate the desired electrode potential as shown in Supplementary Table 7.

## Supplementary information

Supplementary Information

Author Checklist

## Data Availability

The data that support the findings of this study are available from the authors upon request.
