# Supplementary material for: Promoting ethylene production over a wide potential window on Cu crystallites induced and stabilized via current shock and charge delocalization
Source: Nat Commun. 2021 Nov 24;12:6823. doi: 10.1038/s41467-021-27169-9 (PMC8613262; doi:10.1038/s41467-021-27169-9)
Supplement: Supplementary file 1 — Supplementary Information [file 41467_2021_27169_MOESM1_ESM.pdf]

Supplementary Information

Promoting Ethylene Production Over a Wide Potential Window on Cu Crystallites Induced and Stabilized via Current shock and Charge delocalization

Hao Sun[1,2,5], Ling Chen[3,5], Likun Xiong[1,2], Kun Feng[4], Yufeng Chen[4], Xiang Zhang[1,2], Xuzhou Yuan[1,2], Baiyu Yang[1,2], Zhao Deng[1,2], Yu Liu[1,2], Mark H. Rümmeli[1,2], Jun Zhong[4]*, Yan Jiao[3]*, and Yang Peng[1,2]*

[1]Soochow Institute for Energy and Materials Innovations, College of Energy, Soochow University, Suzhou 215006, P. R. China. [2]Key Laboratory of Advanced Carbon Materials and Wearable Energy Technologies of Jiangsu Province, Suzhou, P. R. China. [3]School of Chemical Engineering and Advanced Materials, The University of Adelaide, Adelaide SA 5005, Australia, [4]Institute of Functional Nano & Soft Materials (FUNSOM), Jiangsu Key Laboratory for Carbon-Based Functional Materials & Devices, Soochow University, Suzhou 215123, China. [5]These authors contributed equally: Hao Sun, Ling Chen. Correspondence and requests for materials should be addressed to J.Z. (email: jzhong@suda.edu.cn) or to Y.J. (yan.jiao@adelaide.edu.au) or to Y.P. (email: ypeng@suda.edu.cn)

Methods

Faradaic Efficiency of Gas Products.

$$FE_a = \frac{\frac{v \times C_a}{A \times V_m} \times Z_a \times F}{j_{total}} \times 100\%$$

FE_a: Faradaic Efficiency of the product a

v: CO_2 gas flow rate (L s^{-1})

C_a: Volumefraction of the product a detected by GC

A: Geometric area of the electrode (cm^{-2})

V_m: molar Volume (22.4 L mol^{-1})

Z_a: electrons transferred for reduction to product a

F: Faradaic Constant (C mol^{-1})

j_{total}: Total current density during CO_2 bulk electrolysis (A cm^{-2})

Partial current density.

$$j_a = FE_a \times j_{total}$$

Figures and Tables

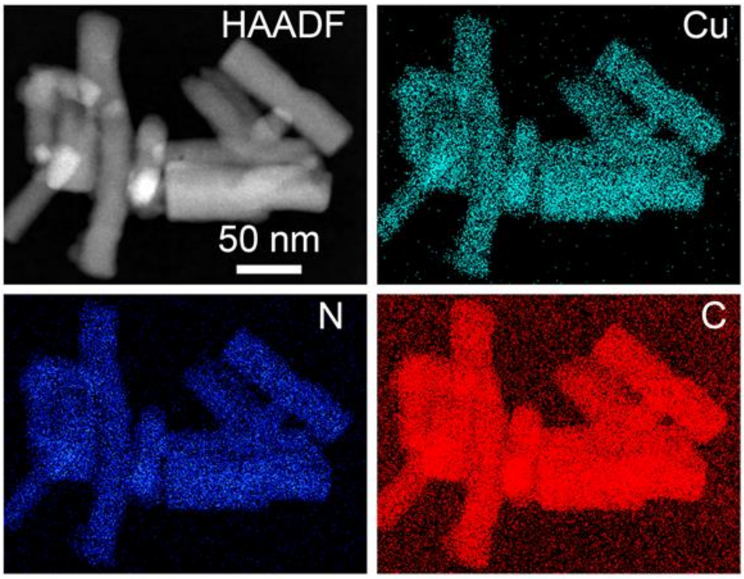

Supplementary Fig. 1 EDX-mapping images of $Cu_3(HITP)_2$ showing the homogeneous distribution of Cu, N, and C.

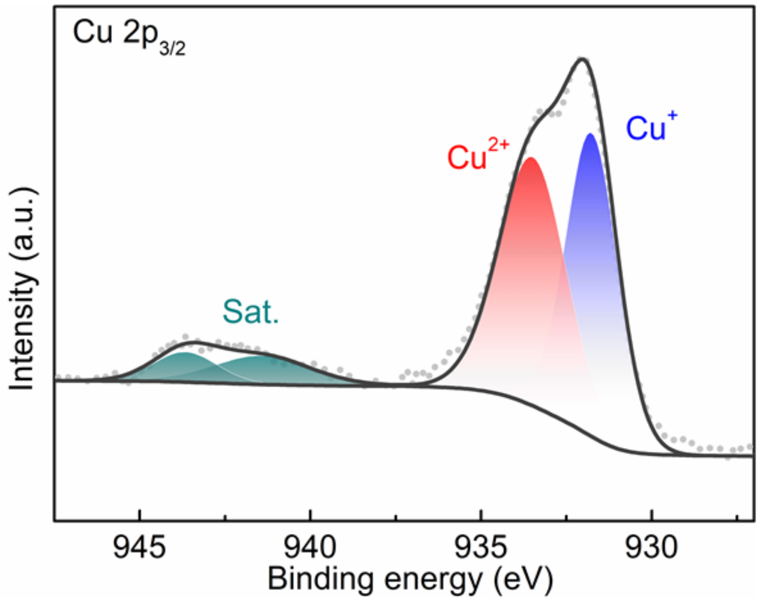

Supplementary Fig. 2 XPS Cu $2p_{3/2}$ spectrum of $Cu_3(HITP)_2$.

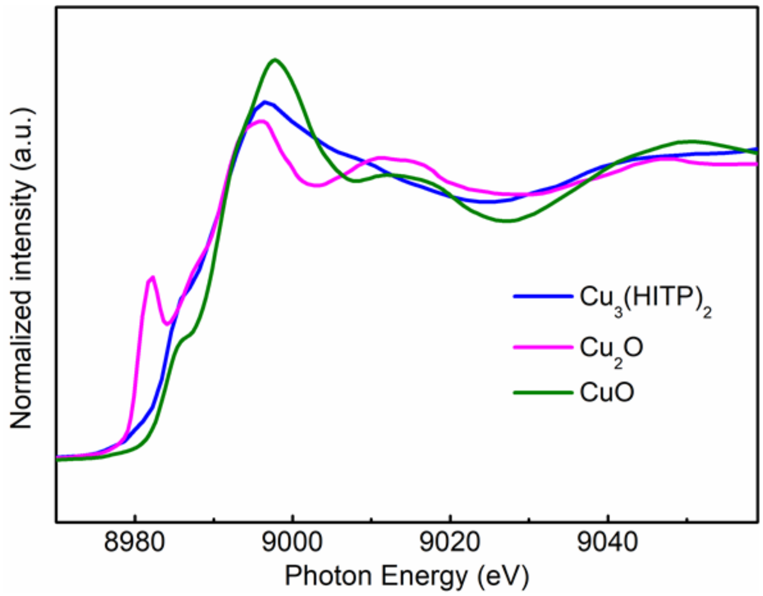

Supplementary Fig. 3 Normalized Cu K-edge XANES spectra of $Cu_3(HITP)_2$ in reference to CuO and Cu_2O.

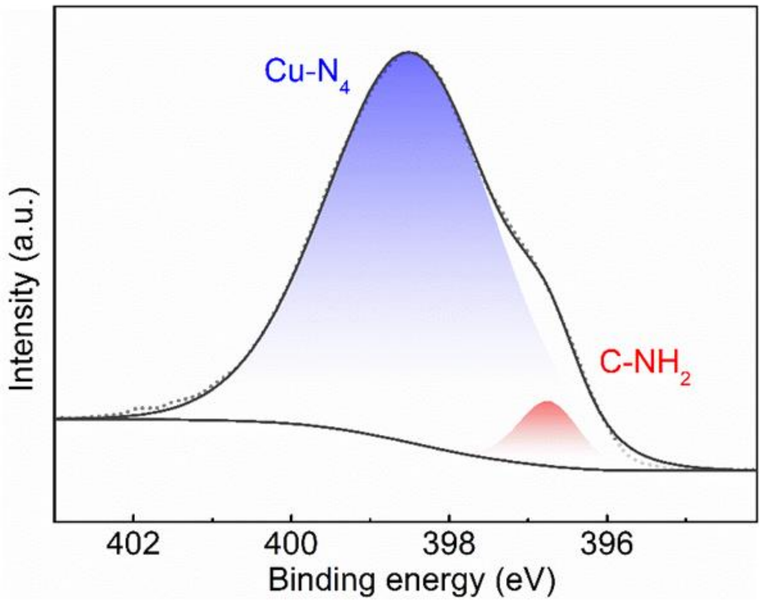

Supplementary Fig. 4 XPS N 1s spectrum of $Cu_3(HITP)_2$.

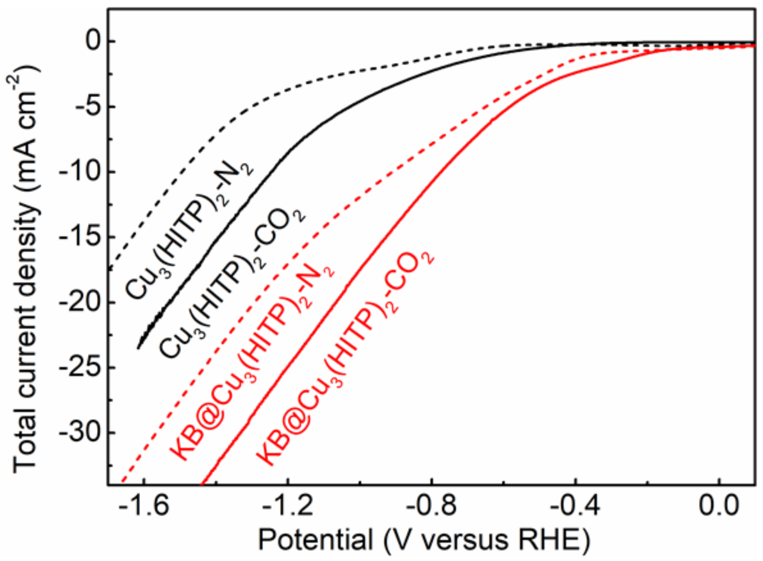

Supplementary Fig. 5 LSV curves of KB@$Cu_3(HITP)_2$ and $Cu_3(HITP)_2$ in N_2/CO_2-saturated 0.1 M $KHCO_3$ solution.

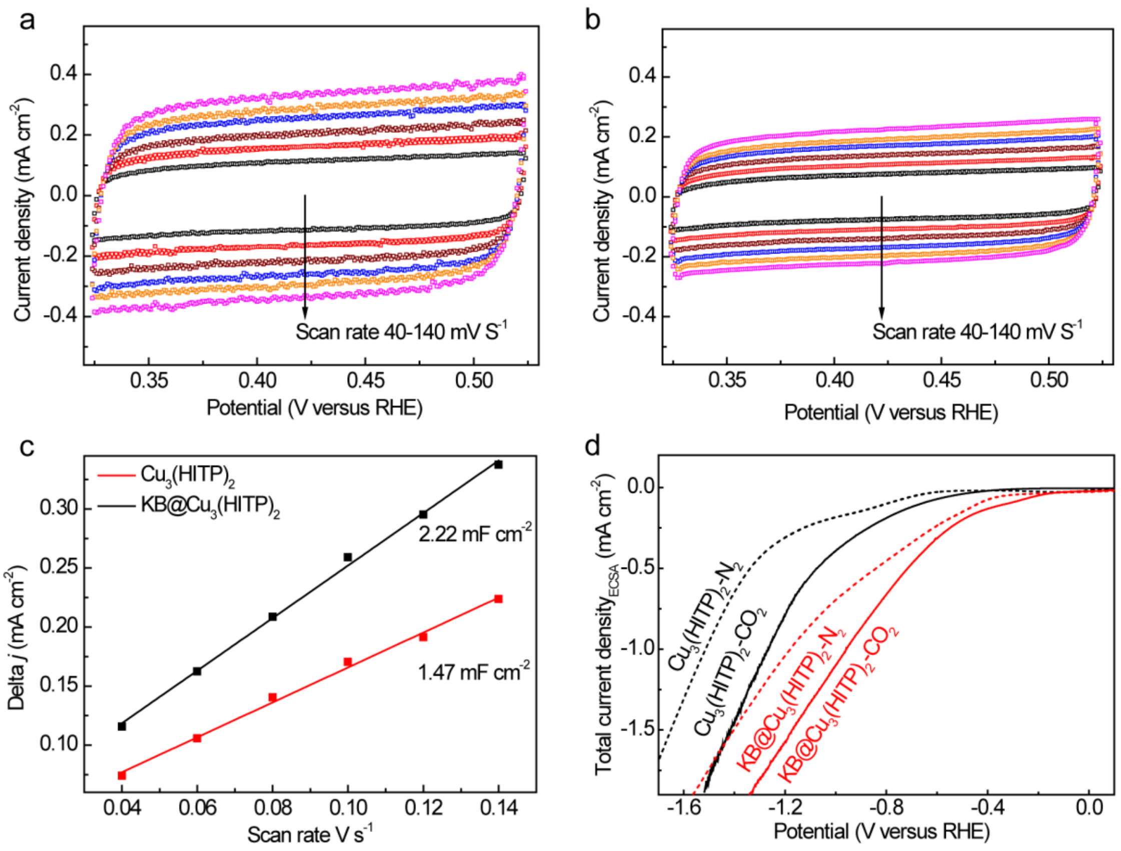

Supplementary Fig. 6 CV curves of (a) KB@$Cu_3(HITP)_2$ and (b) $Cu_3(HITP)_2$ under different scan rates from 40 to 140 mV s^{-1} in 0.1 M $KHCO_3$. (c) The plot of capacitive current at 0.42 V against the scan rate. (d) The LSV curves normalized to ECSA for KB@$Cu_3(HITP)_2$ and $Cu_3(HITP)_2$.

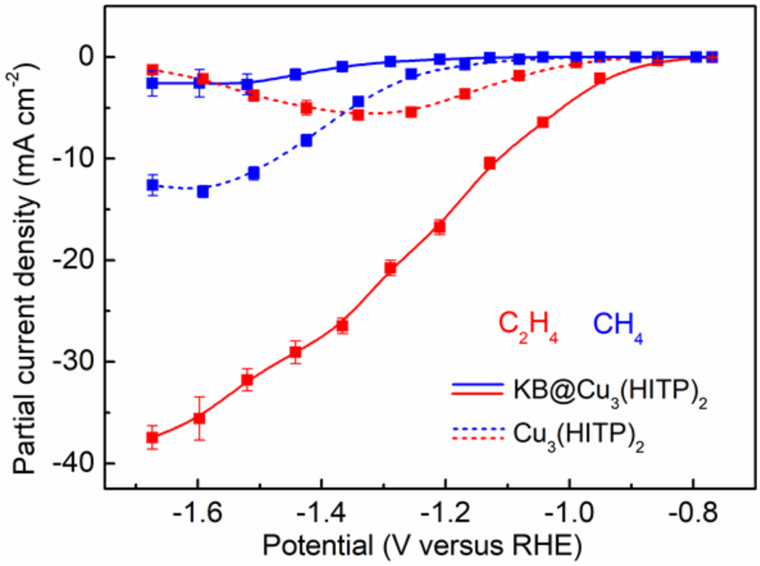

Supplementary Fig. 7 Partial current densities of C_2H_4 and CH_4 production on KB@$Cu_3(HITP)_2$ and $Cu_3(HITP)_2$.

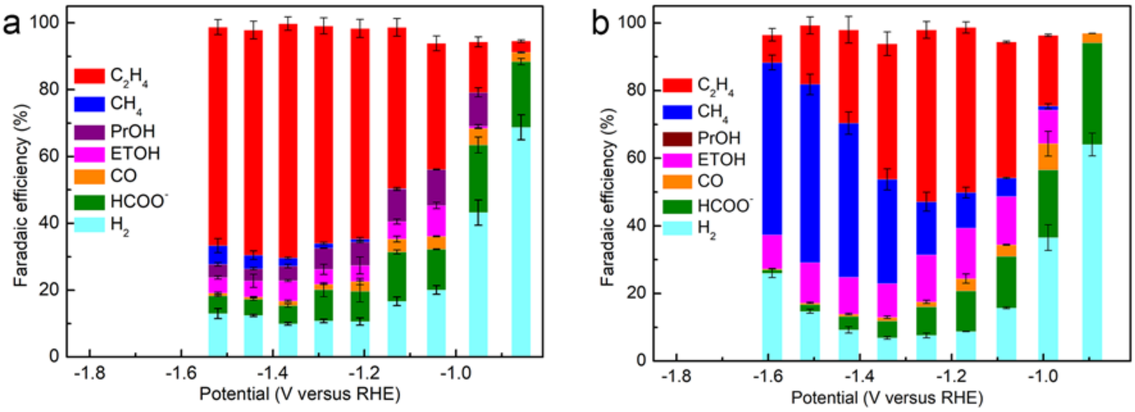

Supplementary Fig. 8 FEs of all CO_2 reduction products for (a) KB@$Cu_3(HITP)_2$ and (b) $Cu_3(HITP)_2$.

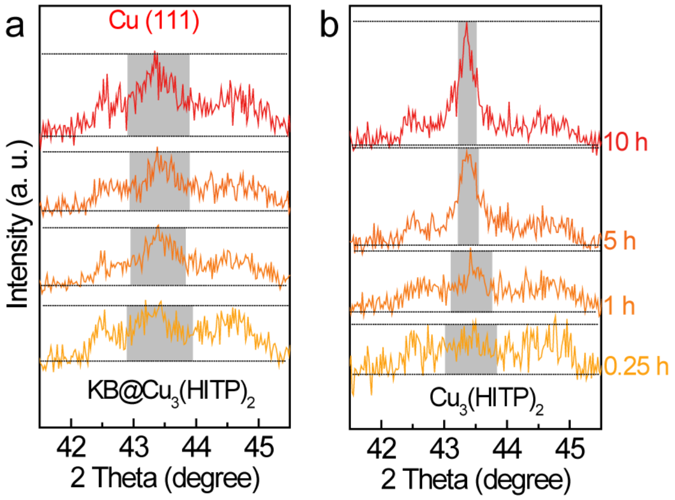

Supplementary Fig. 9 Zoom-in diffractograms for XRD Cu(111) peaks of (a) KB@$Cu_3(HITP)_2$ and (b) $Cu_3(HITP)_2$.

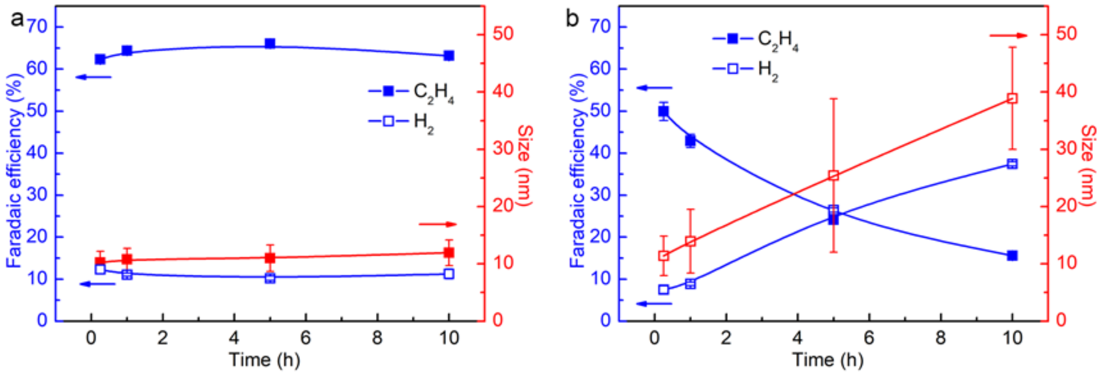

Supplementary Fig. 10 Correlation between the Cu particle size observed in TEM and the FEs of C_2H_4 and H_2 for (a) KB@$Cu_3(HITP)_2$ and (b) $Cu_3(HITP)_2$ in chronoamperometric tests.

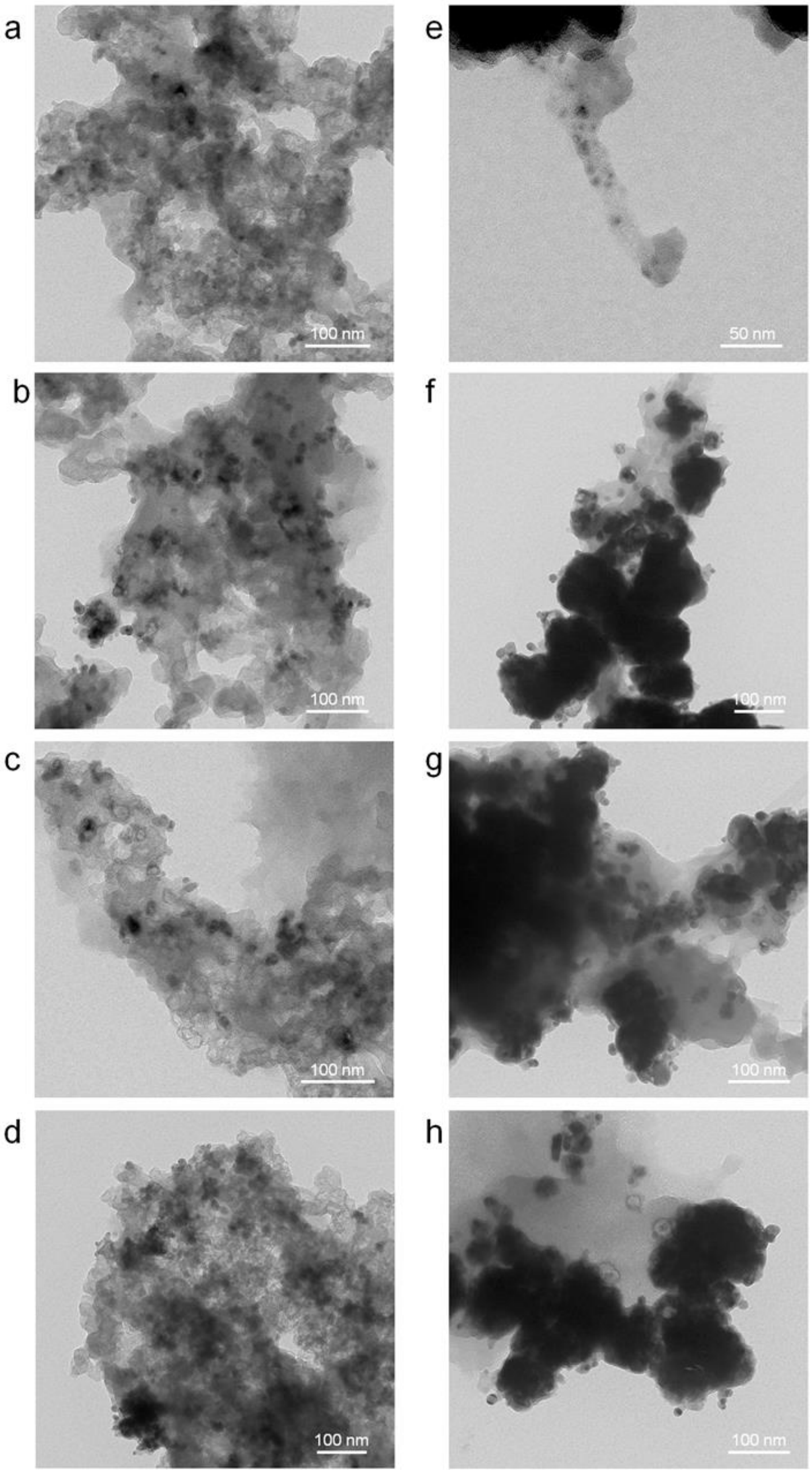

Supplementary Fig. 11 TEM images of KB@$Cu_3(HITP)_2$ after 0.25 h CO_2RR at (a)-1.21 V, (b)-1.37 V, (c)-1.52 V and (d)-1.67 V. TEM images of $Cu_3(HITP)_2$ after 0.25 h CO_2RR at (e)-1.25 V, (f)-1.42 V, (g)-1.59 V and (h)-1.75 V.

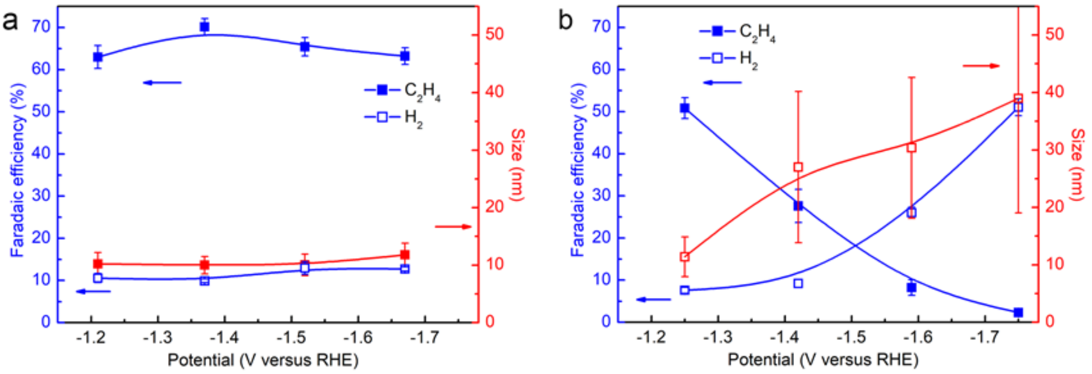

Supplementary Fig. 12 Correlation between the Cu particle size observed in TEM and the FEs of C_2H_4 and H_2 for (a) KB@Cu_3(HITP)$_2$ and (b) Cu_3(HITP)$_2$ at varying potentials.

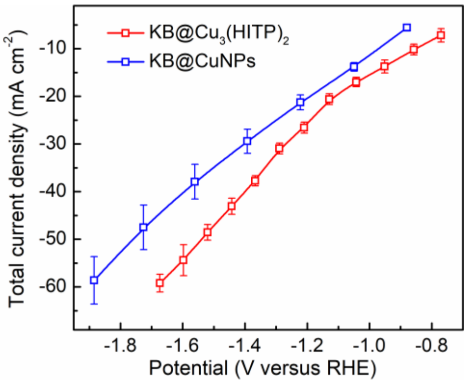

Supplementary Fig. 13 Total current densities on KB@Cu_3(HITP)$_2$ and KB@CuNPs.

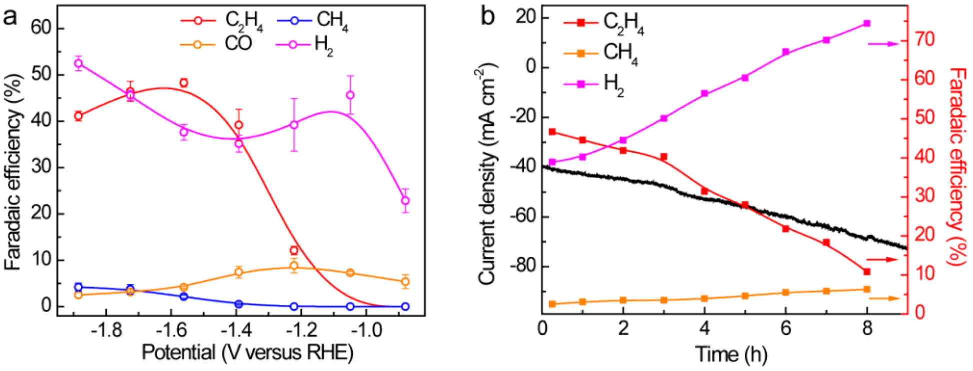

Supplementary Fig. 14 (a) FEs of C_2H_4, CH_4, CO, and H_2 at different potentials tested in a H-cell with 0.1 M $KHCO_3$ for KB@Cu_3(HITP)$_2$ and KB@CuNPs. (b) The chronoamperometric i-t test at −1.56 V for KB@CuNPs showing the evolution of total current density and FEs of C_2H_4, CH_4, and H_2.

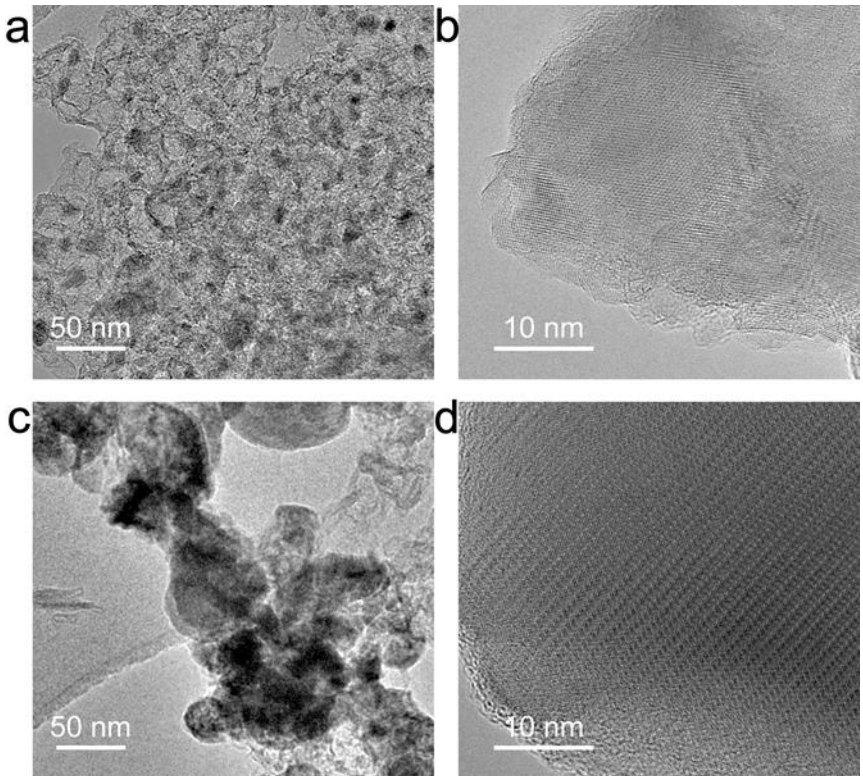

Supplementary Fig. 15 TEM images taken on KB@CuNPs (a, b) before and (c, d) after a chronoamperometric testing period of 8 h at −1.56 V.

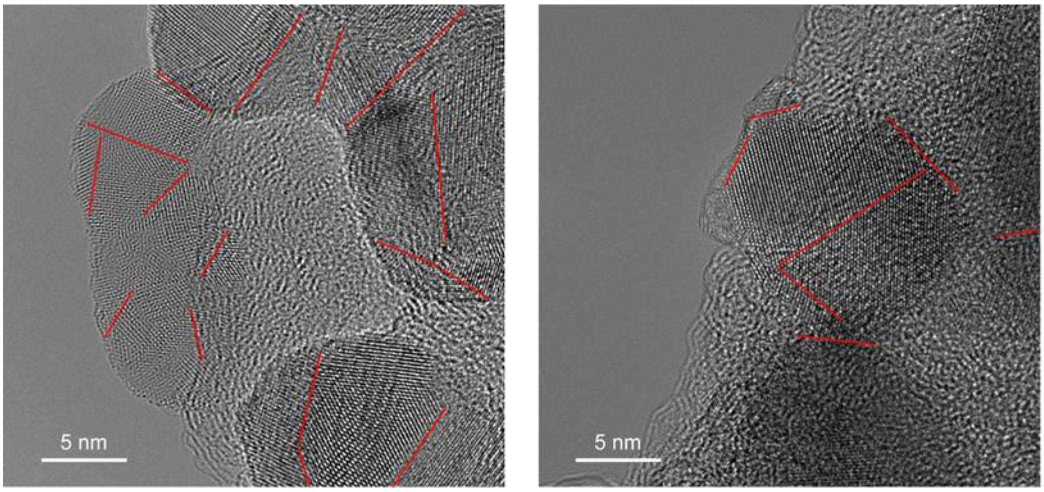

Supplementary Fig. 16 TEM images of KB@$Cu_3(HITP)_2$ after the CO_2RR conducted at -1.25 V for 10 h in CO_2-saturated 0.1 M $KHCO_3$ electrolyte, the lines indicate the grain boundaries.

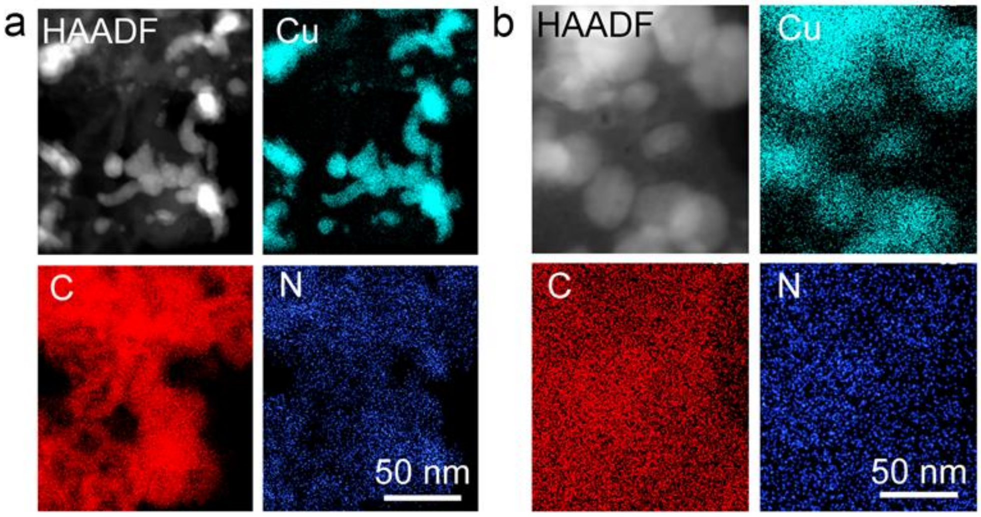

Supplementary Fig. 17 STEM and EDX-Mapping images of (a) KB@$Cu_3(HITP)_2$ and (b) $Cu_3(HITP)_2$ after the CO_2RR conducted at -1.25 V for 10 h in CO_2-saturated 0.1 M $KHCO_3$ electrolyte.

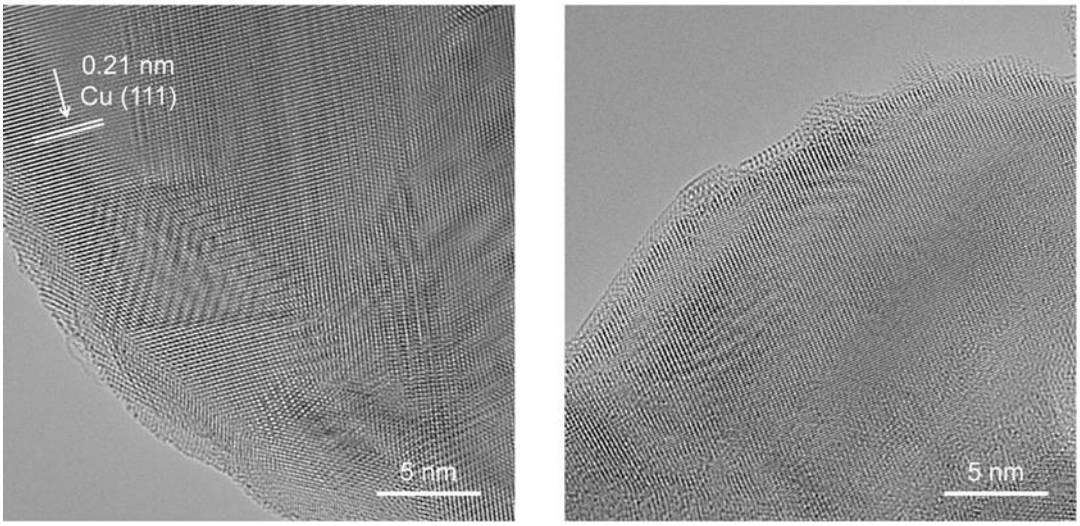

Supplementary Fig. 18 TEM images of $Cu_3(HITP)_2$ after the CO_2RR conducted at -1.25 V for 10 h in CO_2-saturated 0.1 M $KHCO_3$ electrolyte.

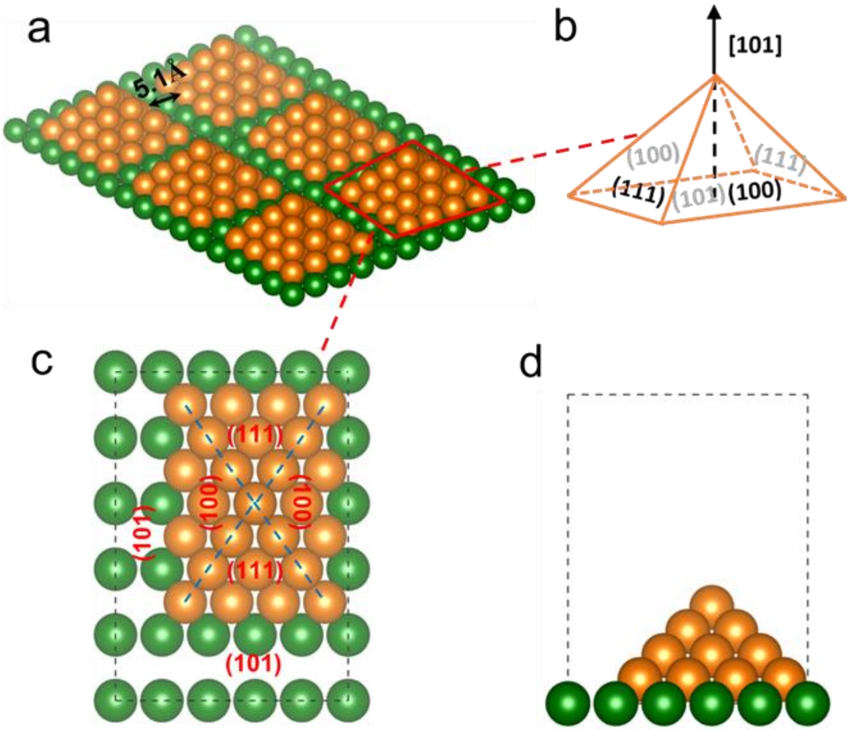

Supplementary Fig. 19 (a) Atomic structures of [101] Cu rectangular nanopyramid to model post-electrolytic KB@$Cu_3(HITP)_2$. (b) Schematic illustration, (c) top view and (d) front view of single Cu rectangular nanopyramid sitting on 5×5×1 Cu(101) supercell. Colour codes: Cu, orange and green. The unit cell is marked with black slash lines and the grain boundaries are marked with blue slash lines as the visual guide.

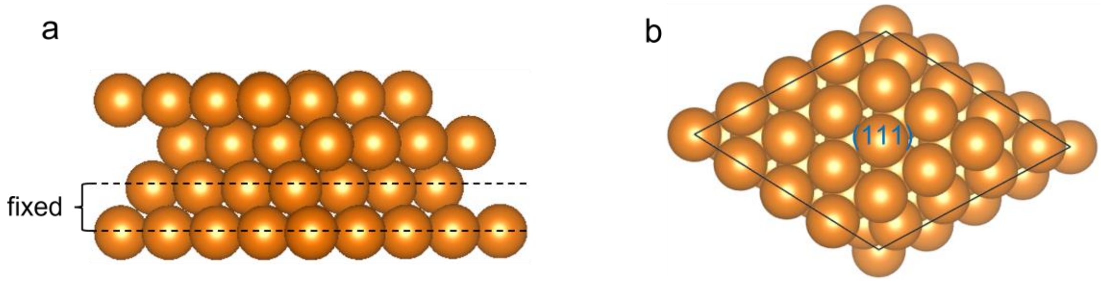

Supplementary Fig. 20 (a) Front view and (b) top view of atomic structures of post-electrolytic $Cu_3(HITP)_2$ based on 4×4×4 Cu(111) supercell. Colour codes: Cu, orange.

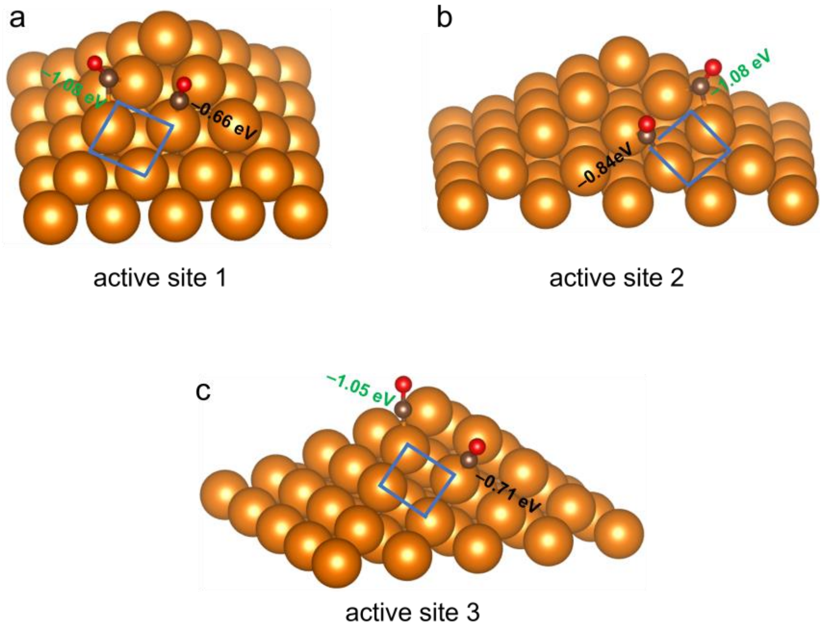

Supplementary Fig. 21 Active sites identified on Cu-RNP surface and the application of *'extended square principle'*[1], i.e. C–C coupling prefers to proceeding between strongly-bound *CO and weakly-bound *CO within under-coordinated (i.e. lattice boundary) surface square sites. E*CO values are marked in green and black, denoting strong and weak adsorption, respectively.

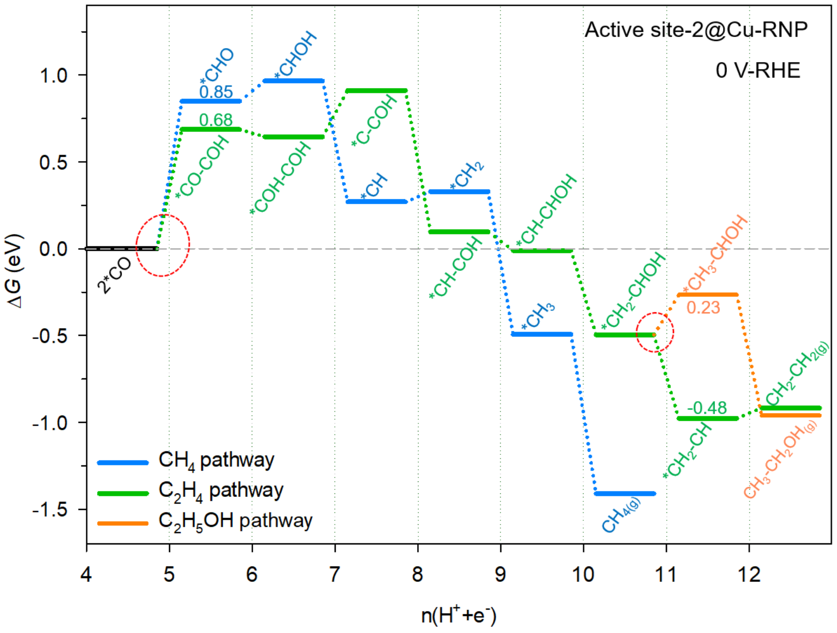

Supplementary Fig. 22 Reaction pathways starting with 2*CO on active site-2 of Cu [101] rectangular nanopyramids (Cu-RNP) at 0 V vs RHE. The key bifurcating points are highlighted with red circles and the unit of energy is eV.

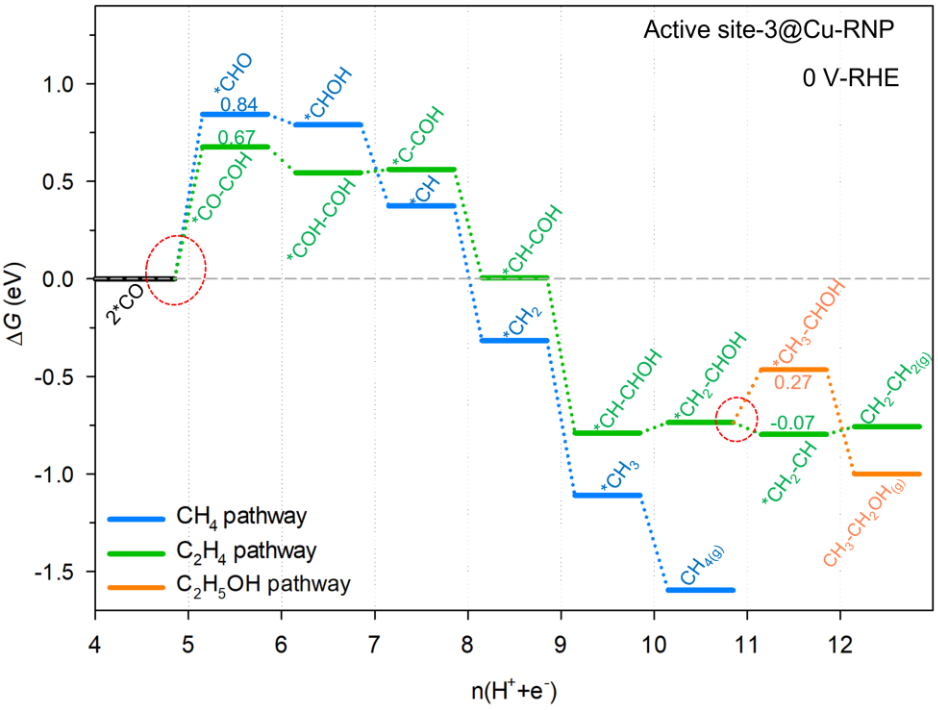

Supplementary Fig. 23 Reaction pathways starting with 2*CO on active site-3 of Cu [101] rectangular nanopyramids (Cu-RNP) at 0 V vs RHE. The key bifurcating points are highlighted with red circles and the unit of energy is eV.

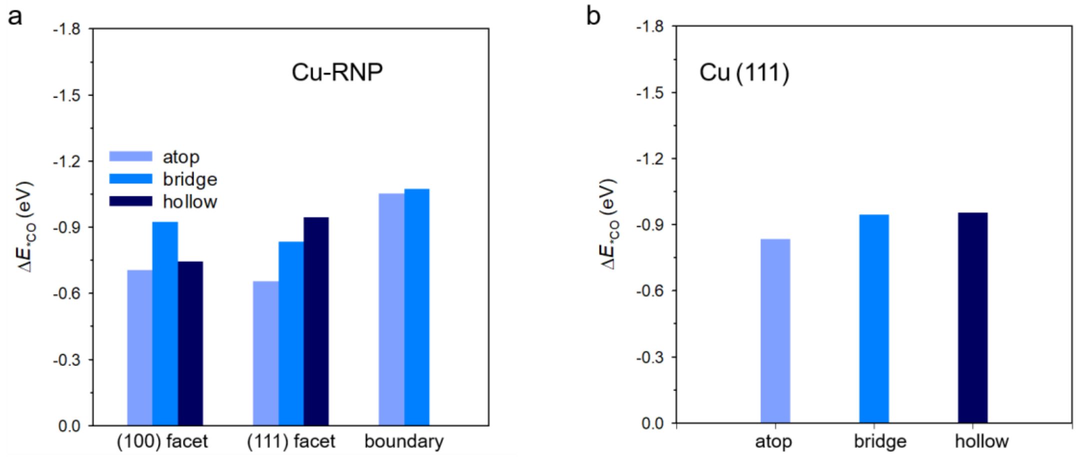

Supplementary Fig. 24 *CO binding energies on various adsorption sites of (a) Cu-RNP and (b) Cu(111).

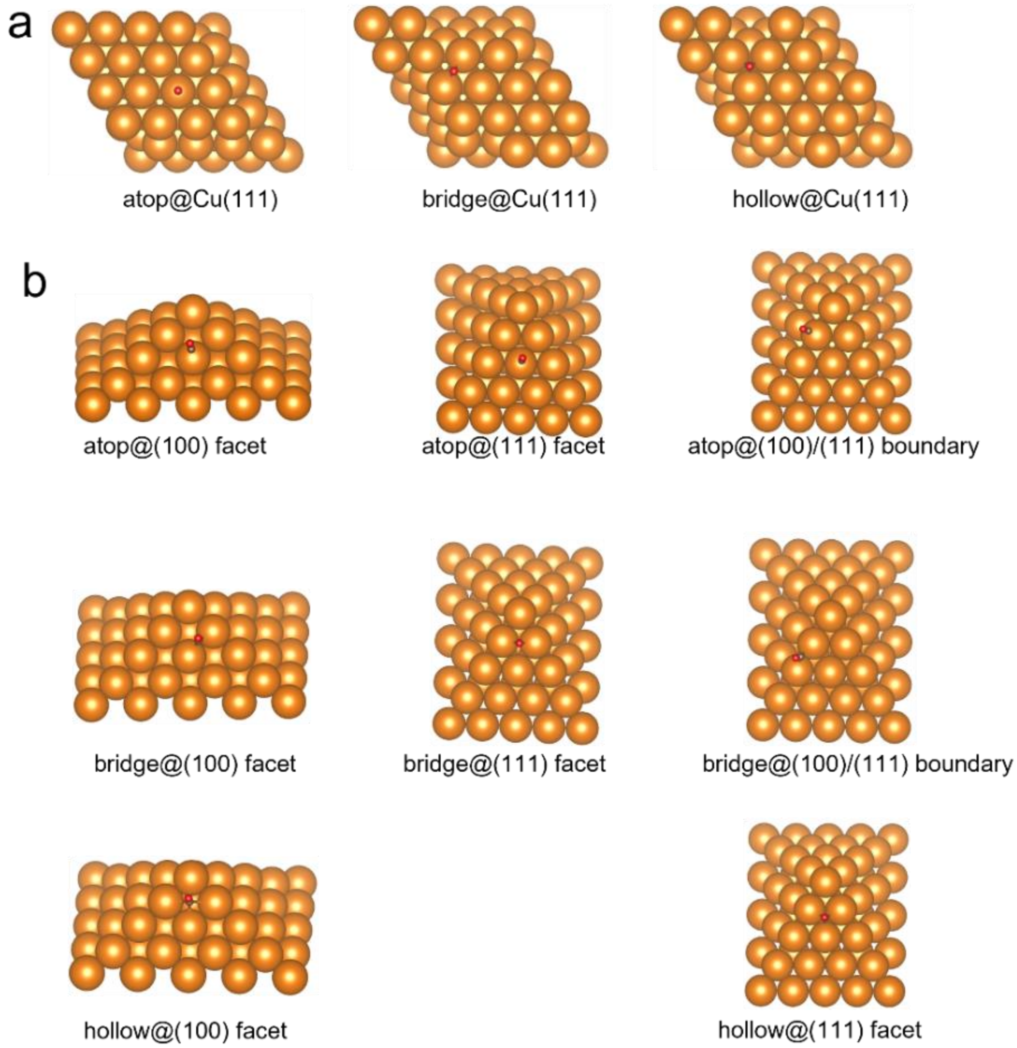

Supplementary Fig. 25. *CO and *H adsorption sites identified on (a) Cu (111) surface and (b) Cu-RNP surface. *CO was used as the exampled atomic configuration.

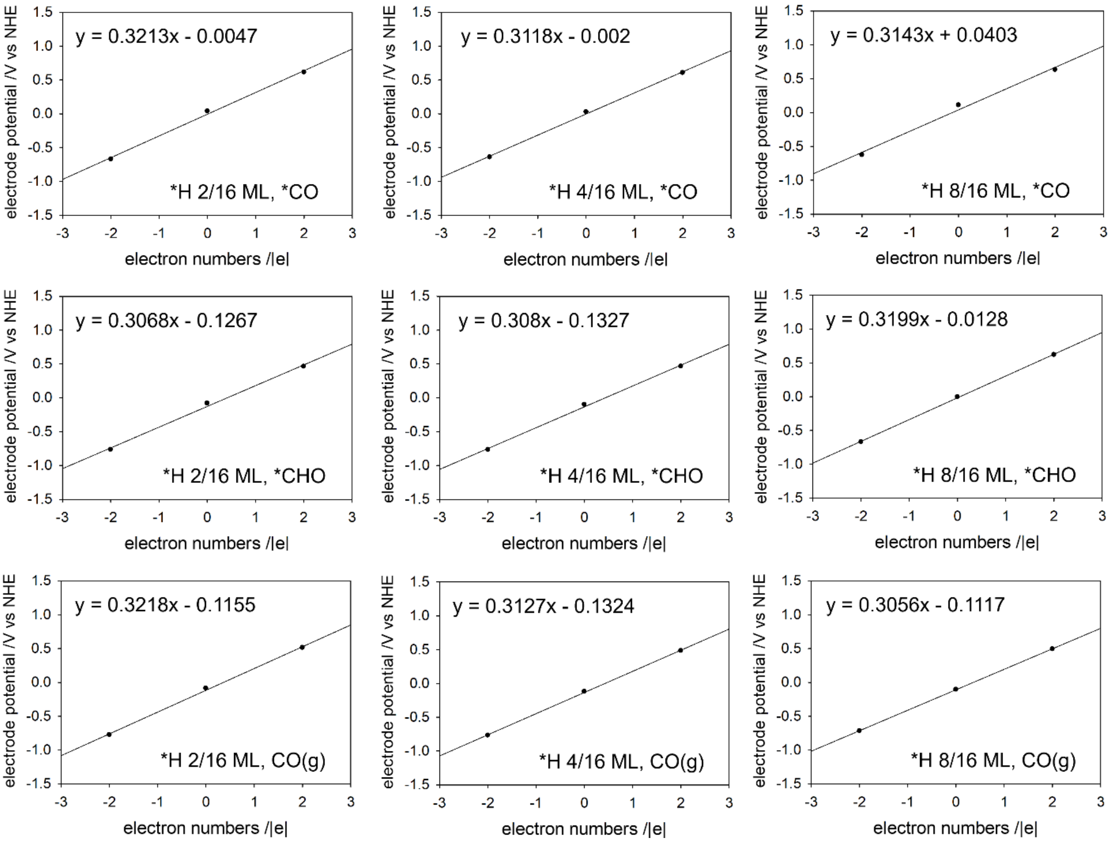

Supplementary Fig. 26. Calculated electrode potentials as the function of surface electron numbers for the intermediates *CO, *CHO and $CO_{(g)}$ on Cu(111) under various *H coverage.

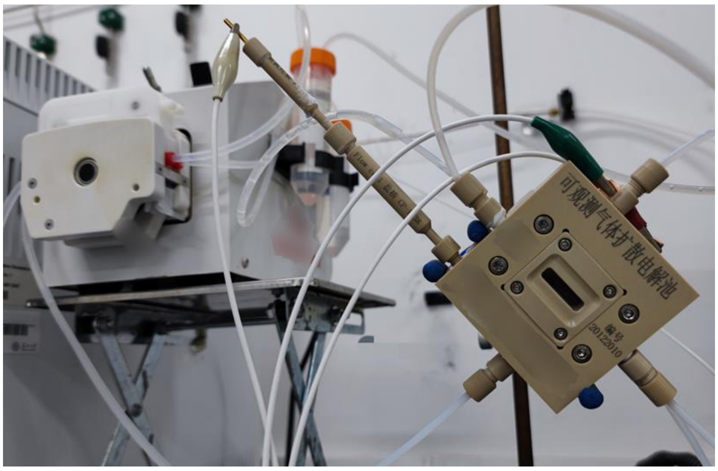

Supplementary Fig. 27 Photograph of the flow cell used for electrocatalytic CO_2RR.

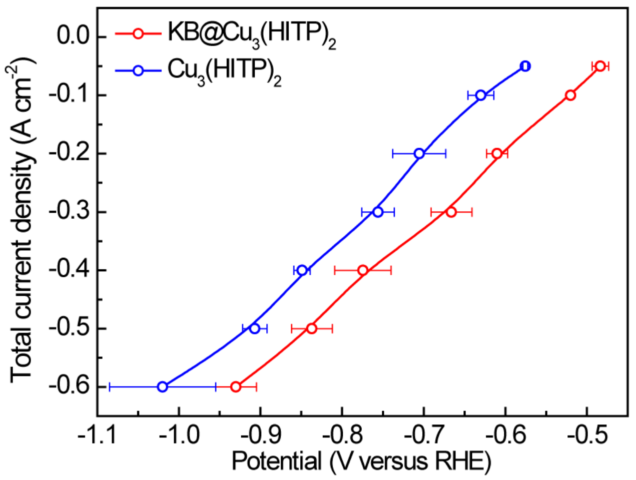

Supplementary Fig. 28 Total current densities on KB@$Cu_3(HITP)_2$ and $Cu_3(HITP)_2$.

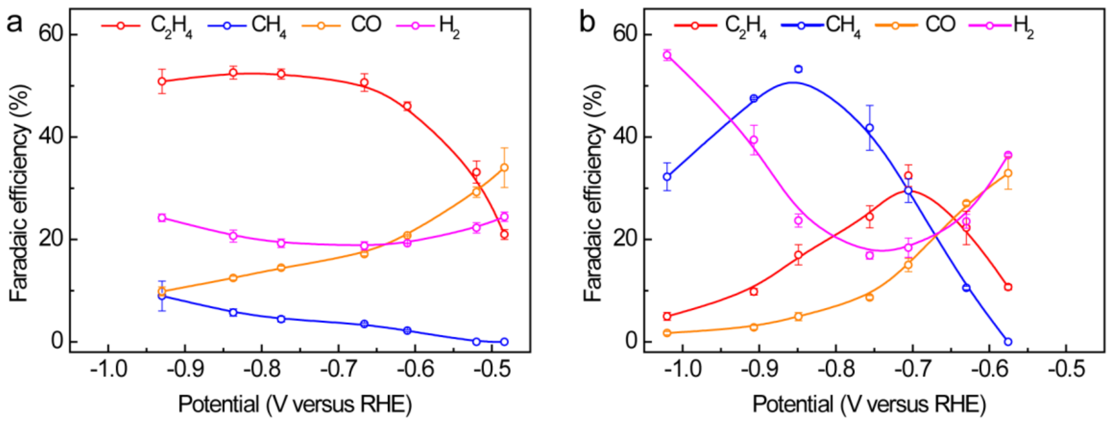

Supplementary Fig. 29 FEs of C_2H_4, CH_4, CO, and H_2 at different potentials tested in a flow-cell with 1 M KOH electrolyte for (a) KB@$Cu_3(HITP)_2$ and (b) $Cu_3(HITP)_2$.

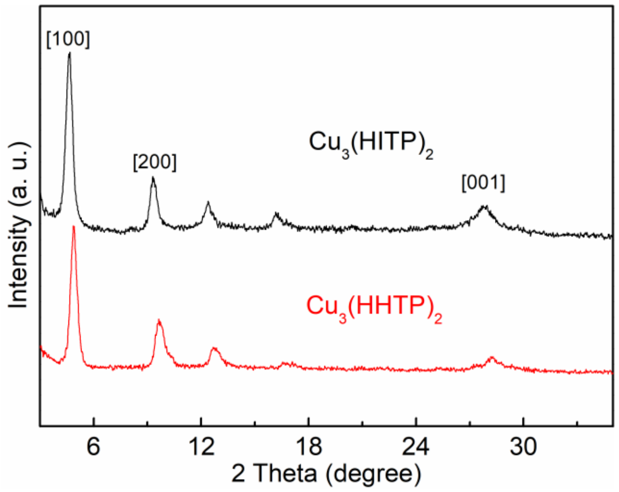

Supplementary Fig. 30 XRD patterns of $Cu_3(HITP)_2$ and $Cu_3(HHTP)_2$.

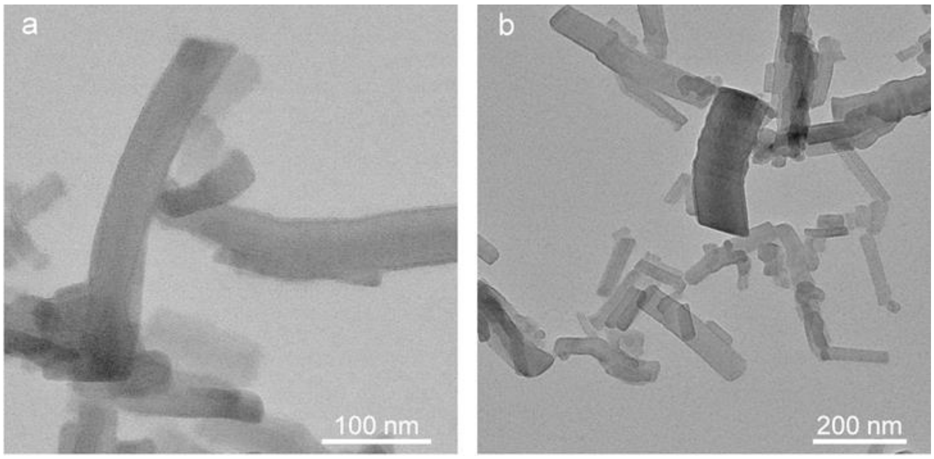

Supplementary Fig. 31 TEM images of $Cu_3(HHTP)_2$.

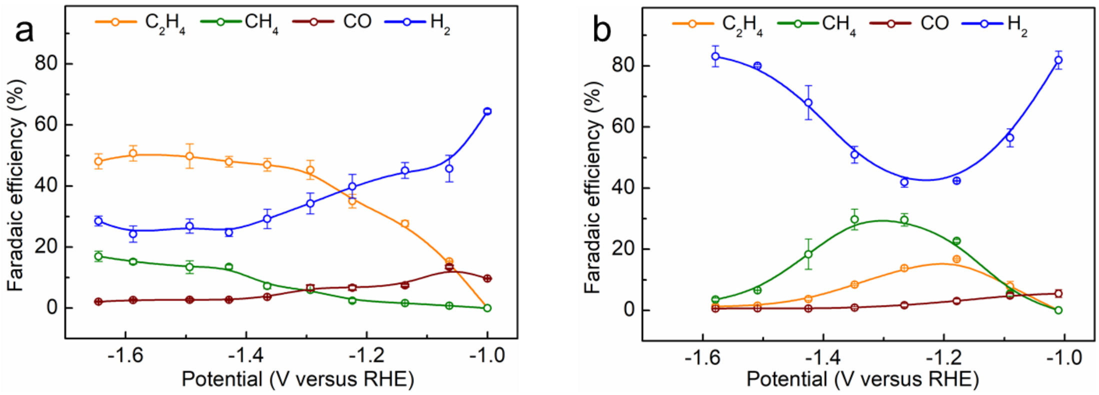

Supplementary Fig. 32 Faradaic efficiencies of CO_2 reduction products for (a) KB@$Cu_3(HHTP)_2$ and (b) $Cu_3(HHTP)_2$ at various potentials.

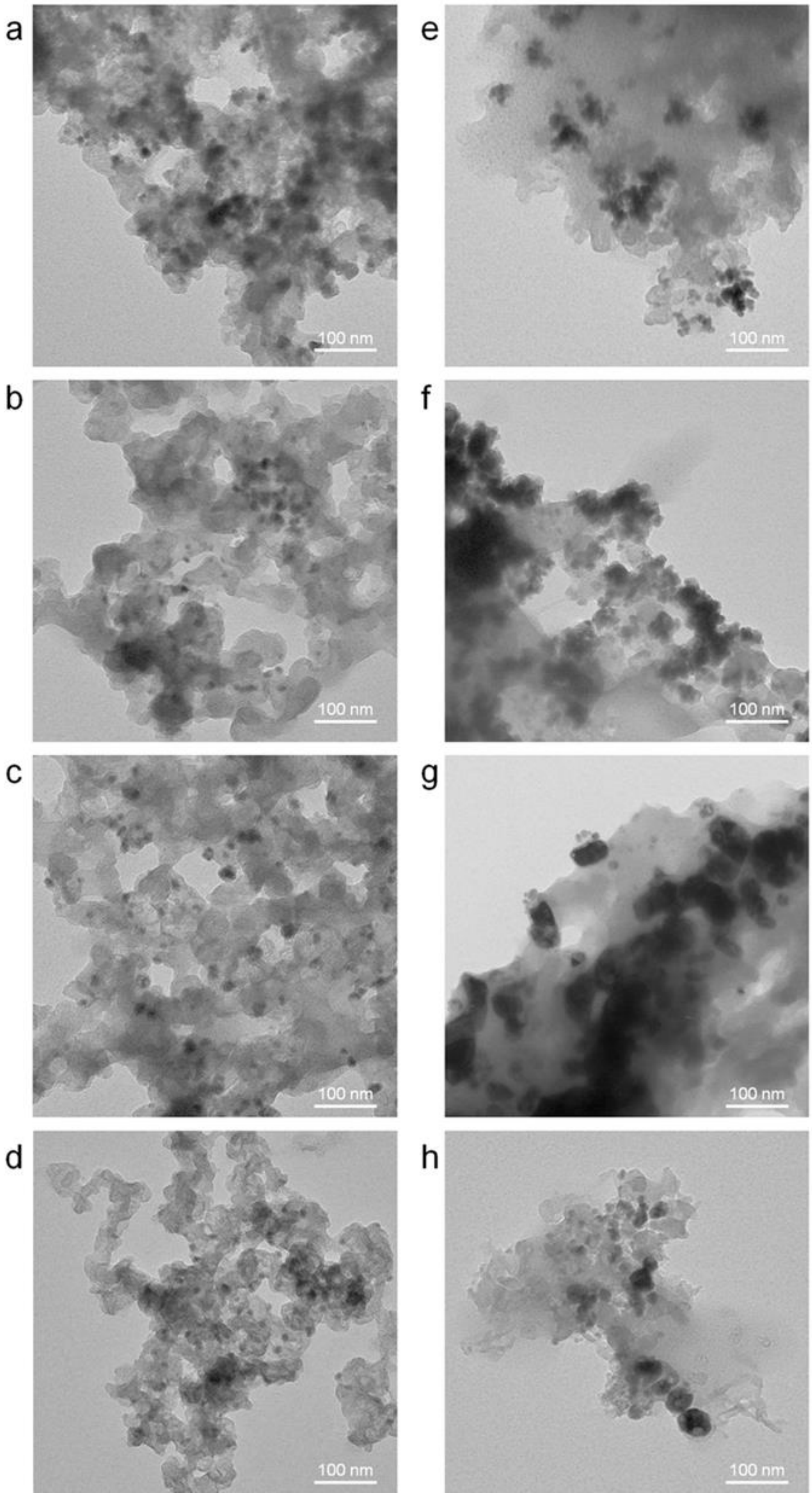

Supplementary Fig. 33 TEM images of KB@$Cu_3(HHTP)_2$ after 0.25 h CO_2RR at (a)-1.22 V, (b)-1.37 V, (c)-1.49 V and (d)-1.65 V. TEM images of KB@$Cu_3(HHTP)_2$ after 0.25 h CO_2RR at (e)-1.26 V, (f)-1.42 V, (g)-1.51 V and (h)-1.73 V.

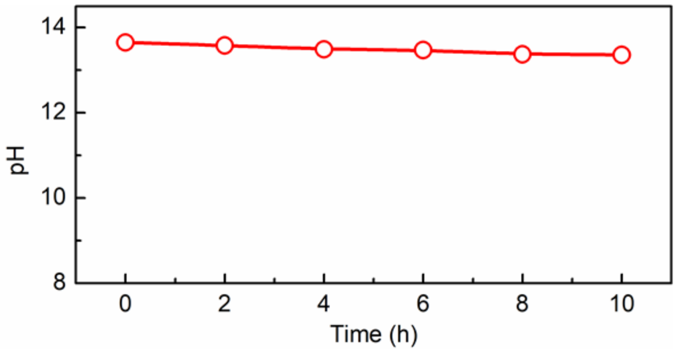

Supplementary Fig. 34 The pH of the electrolyte (1 M KOH) at different reaction time.

Supplementary Table 1. Comparison of CO_2RR performance for KB@$Cu_3(HITP)_2$ and other Cu-based catalysts reported in literature (H-cell).

catalyst	electrolyte	potential (V vs. RHE)	C_2H_4 FE (%)	C_2H_4 j (mA cm^{-2})
KB@$Cu_3(HITP)_2$ (This work)	0.1 M $KHCO_3$	-1.37	~70	~26.5
		-1.67	~64	~37.4
PcCu-Cu-O[2]	0.1 M $KHCO_3$	-1.2	~50	7.3
AN-Cu[3]	0.1 M $KHCO_3$	-1.08	~38.1	~7.3
Cu_2O film[4]	0.1 M $KHCO_3$	-0.99	~37.5	~12.9
Cu-on-Cu_3N[5]	0.1 M $KHCO_3$	-0.95	~39	~14 (C_{2+})
O_2-plasma Cu[6]	0.1 M $KHCO_3$	-0.9	~60	~6.6
ERD Cu[7]	0.1 M $KHCO_3$	-1.2	~38	~22
Cu nanocube[8]	0.25 M $KHCO_3$	-0.96	~32.5	~21
decahedron Cu[9]	0.1 M $KHCO_3$	-0.993	~52	~17.6
Cu-mesocrystal[10]	0.1 M $KHCO_3$	-0.99	~27.2	~6.8
Cu_3-Ag_3Au NFs[11]	0.1 M $KHCO_3$	-1.2	~69	~13
OBC[12]	0.5 M $KHCO_3$	-1.00	45	44.7

Supplementary Table 2. The average size of Cu nanoparticles calculated using the Debye-Scherrer equation.

	0.25 h	1 h	5 h	10 h
KB@$Cu_3(HITP)_2$	15.5 nm	16.0 nm	17.4 nm	14.5 nm
$Cu_3(HITP)_2$	18.7 nm	23.4 nm	46.2 nm	52.3 nm

Debye-Scherrer formula: $\mathrm{D} = \frac{K \times \gamma}{B \times \cos\theta}$

D: average size (nm)
K: Scherrer constant (0.89)
γ: X-ray wavelength (0.154056 nm)
B: FWHM of diffraction peak (rad)
θ: Bragg diffraction Angle (rad)

Supplementary Table 3 Free energy change of all PCET steps along C_2 pathway after 2*CO adsorption on active sites of Cu-RNP and Cu(111). The energy unit is eV.

PCET	Reaction	Active site	ΔG
5th	$*CO+*CO + H^+ + e^- \rightarrow *CO–COH$	1@Cu-RNP	0.61
		2@Cu-RNP	0.68
		3@Cu-RNP	0.67
		Cu(111)	1.30
6th	$*CO–COH + H^+ + e^- \rightarrow *COH–COH$	1@Cu-RNP	0.05
		2@Cu-RNP	–0.04
		3@Cu-RNP	–0.13
7th	$*COH–COH+H^+ + e^- \rightarrow *C–COH + H_2O$	1@Cu-RNP	–0.15
		2@Cu-RNP	0.26
		3@Cu-RNP	0.02
8th	$*C–COH+ H_2O + H^+ + e^- \rightarrow *CH–COH+ H_2O$	1@Cu-RNP	–0.48
		2@Cu-RNP	–0.82
		3@Cu-RNP	–0.56
9th	$*CH–COH+ H_2O + H^+ + e^- \rightarrow *CH–CHOH+ H_2O$	1@Cu-RNP	–0.63
		2@Cu-RNP	–0.11
		3@Cu-RNP	–0.80
10th	$*CH–CHOH+ H_2O + H^+ + e^- \rightarrow *CH_2–CHOH+ H_2O$	1@Cu-RNP	–0.32
		2@Cu-RNP	–0.49
		3@Cu-RNP	0.05
11th	$*CH_2–CHOH+ H_2O + H^+ + e^- \rightarrow *CH_2–CH + 2H_2O$	1@Cu-RNP	–0.19
		2@Cu-RNP	–0.48
		3@Cu-RNP	–0.06
	$*CH_2–CHOH+ H_2O + H^+ + e^- \rightarrow *CH_3–CHOH + H_2O$	1@Cu-RNP	0.26
		2@Cu-RNP	0.23
		3@Cu-RNP	0.27
12th	$*CH_2–CH+ 2H_2O + H^+ + e^- \rightarrow CH_2–CH_2 + 2H_2O$	1@Cu-RNP	0.06
		2@Cu-RNP	0.15
		3@Cu-RNP	0.04
	$*CH_3–CHOH+ H_2O + H^+ + e^- \rightarrow CH_3–CH_2OH + H_2O$	1@Cu-RNP	–0.53
		2@Cu-RNP	–0.70
		3@Cu-RNP	–0.56

Supplementary Table 4. Free energy change of all PCET steps along C1 pathway after 2*CO adsorption on active sites of Cu-RNP and Cu(111). The energy unit is eV.

PCET	Reaction	Active site	Δ*G*
5th	$*CO+*CO + H^+ + e^- \rightarrow *CO + *CHO$	1@Cu-RNP	0.92
		2@Cu-RNP	0.85
		3@Cu-RNP	0.84
		Cu(111)	1.09
6th	$**CO + *CHO + H^+ + e^- \rightarrow *CO + *CHOH$	1@Cu-RNP	0.03
		2@Cu-RNP	0.12
		3@Cu-RNP	–0.06
7th	$*CO + *CHOH+H^+ + e^- \rightarrow *CO +*CH + H_2O$	1@Cu-RNP	–0.66
		2@Cu-RNP	–0.70
		3@Cu-RNP	–0.42
8th	$*CO +*CH + H_2O + H^+ + e^- \rightarrow *CO + *CH_2 + H_2O$	1@Cu-RNP	–0.03
		2@Cu-RNP	0.06
		3@Cu-RNP	0.69
9th	$*CO +*CH_2 + H_2O + H^+ + e^- \rightarrow *CO + *CH_3 + H_2O$	1@Cu-RNP	–0.24
		2@Cu-RNP	–0.82
		3@Cu-RNP	–0.79
10th	$*CO +*CH_3 + H_2O + H^+ + e^- \rightarrow *CO + CH_4 + H_2O$	1@Cu-RNP	–1.39
		2@Cu-RNP	–0.92
		3@Cu-RNP	–0.49

Supplementary Table 5. Comparison of CO2RR performance for Cu3(HITP)2 and other Cu(111) based catalysts reported in literatures.

catalyst	active surface	potentials	CH_4 FE	CO FE
Cu_{oh}[13]	Cu (111)	-0.93 V. vs. RHE	~36.1%	~5.8%
Copper Single Crystal Electrodes[14]	Cu (111)	-1.52 V vs SHE	~50.5%	~4.9%
$Cu_3(HITP)_2$ (This work)	Cu (111)	-1.3 V. vs. RHE	~28%	~2%

Supplementary Table 6. Free energy for gas phase species.

	E *(eV)*	ZPE *(eV)*	–TS *(eV)*	G *(eV)*
H_2 (g)	–6.75	0.27	–0.41	–6.89
H_2O (g)	–14.22	0.56	–0.67	–14.33
CO (g)	–14.80	0.13	–0.61	-14.70
CO_2 (g)	–23.01	0.31	–0.66	–23.36
CH_4 (g)	-24.01	1.19	-0.57	-23.39
C_2H_4 (g)	–31.97	1.37	–0.55	–31.15
C_2H_5OH (g)	–46.88	2.13	–0.60	–45.35

Supplementary Table 7. Electron assigned for the intermediates *CO, *CHO and $CO_{(g)}$ on Cu(111) under different *H coverage to simulate targeted bias potentials.

**Intermediates**	**Potentials**	***H coverage (ML)**	**Electron Assigned (|e|)**
*CO	0 V vs RHE (-0.39 V. vs. NHE)	2/16	-1.20
		4/16	-1.24
		8/16	-1.37
	-1.3 V vs RHE (-1.69 V. vs. NHE)	2/16	-5.24
		4/16	-5.41
		8/16	-5.50
*CHO	0 V vs RHE (-0.39 V. vs. NHE)	2/16	-0.86
		4/16	-0.84
		8/16	-1.18
	-1.3 V vs RHE (-1.69 V. vs. NHE)	2/16	-5.10
		4/16	-5.06
		8/16	-5.24
$CO_{(g)}$	0 V vs RHE (-0.39 V. vs. NHE)	2/16	-0.85
		4/16	-0.82
		8/16	-0.91
	-1.3 V vs RHE (-1.69 V. vs. NHE)	2/16	-4.89
		4/16	-4.98
		8/16	-5.16

References:

1 Chen, L., Tang, C., Jiao, Y. & Qiao, S. Z. Anomalous C-C Coupling on Under-Coordinated Cu (111): A Case Study of Cu Nanopyramids for CO_2 Reduction Reaction by Molecular Modelling. *ChemSusChem* **14**, 671-678, (2021).

2 Qiu, X. F., Zhu, H. L., Huang, J. R., Liao, P. Q. & Chen, X. M. Highly Selective CO_2 Electroreduction to C2H4 Using a Metal-Organic Framework with Dual Active Sites. *J. Am. Chem. Soc.* **143**, 7242-7246, (2021).

3 Lee, S. Y. *et al.* Mixed Copper States in Anodized Cu Electrocatalyst for Stable and Selective Ethylene Production from CO_2 Reduction. *J. Am. Chem. Soc.* **140**, 8681-8689, (2018).

4 Ren, D. *et al.* Selective Electrochemical Reduction of Carbon Dioxide to Ethylene and Ethanol on Copper(I) Oxide Catalysts. *ACS Catal.* **5**, 2814-2821, (2015).

5 Liang, Z. Q. *et al.* Copper-on-nitride enhances the stable electrosynthesis of multi-carbon products from CO_2. *Nat. Commun.* **9**, 3828, (2018).

6 Mistry, H. *et al.* Highly selective plasma-activated copper catalysts for carbon dioxide reduction to ethylene. *Nat. Commun.* **7**, 12123, (2016).

7 De Luna, P. *et al.* Catalyst electro-redeposition controls morphology and oxidation state for selective carbon dioxide reduction. *Nat. Catal.* **1**, 103-110, (2018).

8 Jiang, K. *et al.* Metal ion cycling of Cu foil for selective C–C coupling in electrochemical CO_2 reduction. *Nat. Catal.* **1**, 111-119, (2018).

9 Choi, C. *et al.* A Highly Active Star Decahedron Cu Nanocatalyst for Hydrocarbon Production at Low Overpotentials. *Adv. Mater.* **31**, e1805405, (2019).

10 Chen, C. S. *et al.* Stable and selective electrochemical reduction of carbon dioxide to ethylene on copper mesocrystals. *Catalysis Science & Technology* **5**, 161-168, (2015).

11 Xiong, L. *et al.* Breaking the Linear Scaling Relationship by Compositional and Structural Crafting of Ternary Cu-Au/Ag Nanoframes for Electrocatalytic Ethylene Production. *Angew. Chem. Int. Ed.* **60**, 2508-2518, (2021).

12 Zhang, W. *et al.* Atypical Oxygen-Bearing Copper Boosts Ethylene Selectivity toward Electrocatalytic CO_2 Reduction. *J. Am. Chem. Soc.* **142**, 11417-11427, (2020).

13 De Gregorio, G. L. *et al.* Facet-Dependent Selectivity of Cu Catalysts in Electrochemical CO_2 Reduction at Commercially Viable Current Densities. *ACS Catal.* **10**, 4854-4862, (2020).

14 Hori, Y., Takahashi, I., Koga, O. & Hoshi, N. Selective formation of C_2 compounds from electrochemical reduction of CO_2 at a series of copper single crystal electrodes. *J. Phys. Chem. B* **106**, 15-17, (2002).
