# Supplementary material for: Promoting ethylene production over a wide potential window on Cu crystallites induced and stabilized via current shock and charge delocalization
Source: Nat Commun. 2021 Nov 24;12:6823. doi: 10.1038/s41467-021-27169-9 (PMC8613262; doi:10.1038/s41467-021-27169-9)
Supplement: Supplementary file 2 — Author Checklist [file 41467_2021_27169_MOESM2_ESM.docx]

| 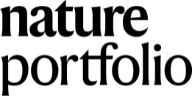 |  |
| --- | --- |
| **Author Checklist** | NCOMMS-NCOMMS-21-28971A |
| **0000000000000000000000000000000000000000000000000000000000000** | **0000000000000000000000000000000000000** |
| **Please check the items below carefully and add a response in each row of the table to indicate the changes that you have made. Please also check through any additional marked-up edits we may have provided within the manuscript file.** | |
|  |  |
|  |  |
|  |  |
| Abstract |  |
| Our guidance: | Your response: |
| When discussing the current work in the abstract, please use the present tense. We also encourage to use active voice | We have used the present tense in the abstract. |
|  |  |
| Author information |  |
| Our guidance: | Your response: |
|  |  |
| Please review your complete author list to verify that it is complete and accurate. We ask that you consult with your coauthors to ensure that all names, affiliations, and titles are represented correctly. Note that if any authors are added or removed after this point then all authors will be requested to provide approval documentation that could potentially delay the production of your paper. | The author list has been confirmed. |
|  |  |
| Article structure |  |
| Our guidance: | Your response: |
| We can accommodate up to 10 display items (Figures or Tables) in the main article. Each Figure and Table must fit easily within an A4 page (210 x 297 mm). Please ensure that the number and size of your Figures and Tables fulfil these requirements to avoid any delay in the acceptance of your article. | Fulfil these requirements. |
| **Please ensure your main manuscript file includes the following sections, in this order:** |  |
| *Title Author list Affiliations Abstract Introduction Results Discussion (optional) Results and Discussion (optional) Methods (including Data Availability, Code Availability and Statistics subsections where relevant) References Acknowledgements Author Contributions Statement Competing Interests Statement Tables Figure Legends/Captions (for main text figures)* |  |
| We do not edit Supplementary Information files; they will be uploaded with the published article as they are submitted with the final version of your manuscript. Any tracked changes should be removed from the file and the file should be provided as a PDF file. Supplementary Figures do not need to be provided separately. | Yes, it has been checked. |
|  |  |
|  |  |
| Main text |  |
| Our guidance: | Your response: |
| Please do not use italics, bold font, underlining or speech marks unless required for technical terms (in both the main text and the display items). | No italics, bold font, underlining or speech marks in the main manuscript. |
| Please make sure that mathematical terms throughout your manuscript and Supplementary Information (including in figures, figure axes, and legends) conform strictly to the following guidelines. Equations must be supplied in editable format, and not as images. Scalar variables (e.g. x, V, χ) must be typeset in italic, whereas multi-letter variables and functions (e.g. log) must be formatted in roman. Vectors (such as the wavevector k or the magnetic field vector B) must be typeset in bold without italics. | Fulfil these requirements. |
| Please label equations sequentially as (1), (2), (3), etc. | Yes. |
| Atomic orbital notations (sp, d, etc.) and corresponding XPS labels should be typeset in italics throughout the main text, figures and Supplementary Information, whereas all accompanying superscripts/subscripts should be typeset in roman font. | It has been confirmed. |
|  |  |
| Figures and Tables |  |
| Our guidance: | Your response: |
| Please see the guidelines linked below for detailed instructions about how your figures should be prepared. Following these instructions will reduce the chances of delays should we need to request replacement artwork from you at a later stage. | OK |
| [https://www.nature.com/documents/NRJs-guide-to-preparing-final-artwork.pdf](https://www.nature.com/documents/NRJs-guide-to-preparing-final-artwork.pdf) |  |
| Please make sure that the terms ‘atomic units (a. u.)’ or ‘arbitrary units (arb. units)’ are appropriately used. | OK |
| Any abbreviations, symbols or colours present in your figures must be defined in the associated legends. | OK |
| Chemical structures in figures should be drawn using the Nature Chemistry template or its settings: http://www.nature.com/authors/guides/NR_chemdraw_stylesheet.cds Refer to the Nature Research Chemical Structures Guide for all details: https://www.nature.com/authors/guides/ChemStructureGuide.pdf | OK |
| In each Figure and Supplementary Figure where error bars are used, they must be defined. | OK |
|  |  |
| Data and Code |  |
| Our guidance: | Your response: |
| Nature journals strongly support public availability of data and code. Please deposit the data and code used in your paper into a public data repository, or alternatively, present the data as Supplementary Information. If data can only be shared on request, please explain why in your Data Availability Statement, and also in the correspondence with your editor.   Please note that for some data types, deposition in a public repository is mandatory. Any restrictions on sharing of these data types must be clearly indicated in the statement and discussed with the editor. More information on our data deposition policies and available repositories can be found here: | OK. |
| [https://www.nature.com/nature-research/editorial-policies/reporting-standards#availability-of-data](https://www.nature.com/nature-research/editorial-policies/reporting-standards#availability-of-data) |  |
| All published manuscripts reporting original research in Nature Portfolio journals must include a data availability statement, as a separate section before the References and under the heading 'Data Availability'.   The data availability statement must make the conditions of access to the “minimum dataset” that are necessary to interpret, verify and extend the research in the article, transparent to readers.   This minimum dataset may be provided through deposition in public community/discipline-specific repositories, custom proprietary repositories or general repositories like Figshare, Zenodo and Dryad. Providing large datasets in supplementary information is strongly discouraged and the preferred approach is to make data available in repositories. Scientific Data, a Nature Portfolio journal, maintains a list of approved and recommended data repositories to support researchers seeking suitable repositories for their data (https://www.nature.com/sdata/policies/repositories).  The Data Availability Statement should also reference any source data published alongside the paper.  If DOIs are provided, we also strongly encourage including these in the Reference list (authors, title, publisher (repository name), identifier, year).  For clinical datasets or third party data, please ensure that the statement adheres to our policy (https://www.nature.com/nature-research/editorial-policies/reporting-standards#availability-of-data) | The data that supports the findings of this study is available from the authors upon the request. |
| Please use the following template to provide all the information stated above:  The XX data generated in this study have been deposited in the YY database under accession code ZZ [add hyperlink here]. The XX data are available under restricted access for {insert reason}, access can be obtained by {explain how}. The raw XX data are protected and are not available due to data privacy laws. The processed XX data are available at YY. The XX data generated in this study are provided in the Supplementary Information/Source Data file. The XX data used in this study are available in the YY database under accession code ZZ [Add hyperlink here]. | The data that supports the findings of this study is available from the Supplementary Information and the authors upon the request. |
|  |  |
|  |  |
| References |  |
| Our guidance: | Your response: |
| Supplementary References should appear at the end of the Supplementary Information file, and must be self-contained and numbered from 1. References mentioned in both the main text and the Supplementary Information should be part of both reference lists so that the Supplementary Information does not refer to the reference list in the main paper and vice versa. | It has been confirmed. |
|  |  |
| End matter |  |
| Our guidance: | Your response: |
| Nature Portfolio defines Competing Interest (CI) as financial and non-financial interests (including but not limited to funding, employment, stocks, shares, patents, personal or professional relationships with individuals or institutions, and unpaid membership advocacy) that could be perceived to directly undermine the objectivity, integrity, and value of a publication, or could be seen as having an influence on the judgments and actions of authors with regard to objective data presentation, analysis, and interpretation.  Please thoroughly review our policy on Competing Interests and include a detailed statement both in your final manuscript file and in our manuscript tracking system. Please ensure the statements are identical in both. Be specific about how each point stated relates to the research and list applicable author initials, and/or patent numbers.  If there are no competing interests, a negative statement must be included. | The authors declare no competing interests. |
| <https://www.nature.com/nature-research/editorial-policies/competing-interests> |  |
| Please confirm that all relevant funding awarded to each author is described in the Acknowledgements section. List each grant number, followed by the initials of the author who received it. | It has been confirmed. |
|  |  |
| Preparing your manuscript files |  |
| Our guidance: | Your response: |
| Unless otherwise stated please limit individual file sizes to approximately 30MB. We strongly encourage the use of repositories for large datasets or source data due to size considerations. | OK |
| In addition, please supply a two sentence summary of your work to accompany the paper on our homepage. The summary should be free from too many abbreviations, accessible to a broad audience, contain no more than 250-300 characters including spaces, and should include two sentences, the first of which describes the background to the work, and the second of which summarises the major conclusions. The summary should be written in the third person in language suitable for a broad audience. The summary may be edited by the editors prior to publication. Please provide this summary in your cover letter. | The summary has been provided in the cover letter. |
| To ensure maximum visibility for your work, we may tweet about your paper following publication. If you would like us to include the Twitter handles of the first author(s), corresponding author(s), lab or institution in this tweet, please provide them in your cover letter. We would also welcome your suggestions for hashtags to use when tweeting about the work. | No |
| Please provide figures as individual vector files with editable text. Acceptable file types for figures are .ai, .eps, .pdf or Chem Draw for fully editable vector-based art. For detailed guidance on figure preparation, see https://www.nature.com/documents/aj-artworkguidelines.pdf | OK |
| Please supply the main Supplementary Information file as a single PDF file. | OK |
| The use or adaptation of previously published images is strongly discouraged. If this is unavoidable, please request the necessary rights documentation to re-use such material from the relevant copyright holders and return this to us when you submit your revised manuscript. Please check whether your manuscript or Supplementary Information contain third-party images, such as figures from the literature, stock photos, clip art or commercial satellite and map data.  For more information on what constitutes ownership by a third party, please contact our Editorial Assistant at naturecommunications@nature.com | It has been confirmed. |
|  |  |
|  |  |
| Forms to complete |  |
| Our guidance: | Your response: |
| **Editorial Policy Checklist** |  |
| Please update and upload a final version of the Editorial Policy Checklist with your revised manuscript files. A blank Editorial Policy Checklist can be found via the link below. Note that this form is a dynamic ‘smart pdf’ and must be downloaded and completed in Adobe Reader. |  |
| Please update your current checklist or download from: | OK |
| [https://www.nature.com/documents/nr-editorial-policy-checklist.zip](https://www.nature.com/documents/nr-editorial-policy-checklist.zip) |  |
|  |  |
|  |  |
| **You will need to upload:** |  |
| Editorial Policy Checklist | √ |
| Completed Third Party Rights Table (if relevant) |  |
| A point-by-point response to the reviewers' comments | √ |
| A completed copy of this checklist | √ |
| The main article file in Microsoft Word format - please supply a version with tracked changes and a version with tracked changes accepted | √ |
| Separate Figure files | √ |
| Inventory of Supporting Information |  |
| A Supplementary Information file | √ |
| ChemDraw files |  |
|  |  |
